# CD8^+^ T cells induce RIPK1-regulated brain endothelial cell death in cerebral malaria

**DOI:** 10.1126/sciadv.aej4381

**Published:** 2026-11-06

**Authors:** Alexandros Hadjilaou, Phaidon Christodoulou, Johannes Brandi, Jan Broder Engler, Ana Matcharashvili, Tatiana Goncharov, Carla Johnson, Nikita Vladimirov, Charlotte Schubert, Sanjib Mohanty, Praveen K. Sahu, Linda Drewicke, Sonja Binder, Oliver Kretz, Susanne Krasemann, Lukas C. Bal, Marcel S. Woo, Sandra Konrath, Dagmara McGuinness, Vanessa Vieira, Ulricke Richardt, Christiane Steeg, Simone Bauer, Terrie Taylor, James Nyirenda, Thomas Renné, Karl B. Seydel, Angelika Hoffmann, Jens Kuhle, Zhen Huang, Peter Vandenabeele, Andrew Woehler, Christopher A. Moxon, Markus Schwaninger, Samuel C. Wassmer, Victor G. Puelles, Domagoj Vucic, Thomas Jacobs, Manuel A. Friese

**Affiliations:** ^1^ Institute of Neuroimmunology and Multiple Sclerosis, University Medical Center Hamburg-Eppendorf, Hamburg, Germany.; ^2^ Protozoa Immunology, Bernhard Nocht Institute for Tropical Medicine (BNITM), Hamburg, Germany.; ^3^ German Center for Infection Research (DZIF), partner site Hamburg-Lübeck-Borstel-Riems, Hamburg, Germany.; ^4^ Hamburg Center for Kidney Health (HCKH), University Medical Center Hamburg-Eppendorf, Hamburg, Germany.; ^5^ III. Department of Medicine, University Medical Center Hamburg-Eppendorf, Hamburg, Germany.; ^6^ Department of Immunology Discovery, Genentech, San Francisco, CA, USA.; ^7^ Center for Parasitology, School of Infection & Immunity, College of Medical, Veterinary and Life Sciences, University of Glasgow, Glasgow, UK.; ^8^ Berlin Institute for Medical Systems Biology, Max Delbrück Center for Molecular Medicine, Berlin, Germany.; ^9^ Brain Research Institute, University of Zurich, Zurich, Switzerland.; ^10^ Department of Molecular Biology and Infectious Diseases, Community Welfare Society Hospital, Rourkela, Odisha, India.; ^11^ Center for the Study of Complex Malaria in India, Ispat General Hospital, Rourkela, Odisha, India.; ^12^ Institute for Experimental and Clinical Pharmacology and Toxicology, Center of Brain, Behavior and Metabolism, University of Lübeck, Lübeck, Germany.; ^13^ Core Facility Electron Microscopy, University Medical Center Hamburg-Eppendorf, Hamburg, Germany.; ^14^ Institute of Neuropathology, University Medical Center Hamburg-Eppendorf, Hamburg, Germany.; ^15^ Institute of Clinical Chemistry and Laboratory Medicine, University Medical Center Hamburg-Eppendorf, Hamburg, Germany.; ^16^ Blantyre Malaria Project, Kamuzu University of Health Sciences, Blantyre, Malawi.; ^17^ Malawi-Liverpool-Wellcome Programme, Kamuzu University of Health Sciences, Blantyre, Malawi.; ^18^ University Institute of Diagnostic and Interventional Neuroradiology, University Hospital Bern, Inselspital, University of Bern, Bern, Switzerland.; ^19^ Department of Neurology, University Hospital Basel, Basel, Switzerland.; ^20^ Departments of Biomedicine and Clinical Research, University of Basel, Basel, Switzerland.; ^21^ Departments of Neuroscience and Neurology, University of Wisconsin-Madison, Madison, WI, USA.; ^22^ VIB Center for Inflammation Research, VIB, Ghent B-9052, Belgium.; ^23^ Department of Biomedical Molecular Biology, Ghent University, Ghent B-9052, Belgium.; ^24^ Department of Infection Biology, London School of Hygiene and Tropical Medicine, London, UK.; ^25^ Department of Clinical Medicine, Aarhus University, Aarhus, Denmark.; ^26^ Department of Pathology, Aarhus University Hospital, Aarhus, Denmark.

## Abstract

Cerebral malaria (CM) is a potentially lethal complication of *Plasmodium falciparum* infection characterized by blood-brain barrier disruption and brain hemorrhage. Although immune-mediated endothelial damage is central to CM pathology, the mechanisms linking cytotoxic immune responses to endothelial injury remain unclear. Here, we show that CD8^+^ T cells induce receptor-interacting serine/threonine-protein kinase 1 (RIPK1)–regulated cell death of brain endothelial cells in mouse CM, while RIPK1 phosphorylation is increased in brain endothelial cells in human CM. Using magnetic resonance imaging, three-dimensional tissue-cleared brain imaging, cross-species pathology, endothelial translatomics, immunocerebrovascular interactomics, and genetic association testing, we identify a CD8^+^ T cell–dependent, RIPK1-centered mouse endothelial cell death program. Combined RIPK1 and caspase inhibition protected cross-presenting endothelial cells from CD8^+^ T cell–mediated death in vitro. In vivo, brain endothelial-specific genetic inactivation or systemic pharmacological inhibition of RIPK1 kinase activity prolonged survival in mouse CM without altering parasitemia. These findings support RIPK1 inhibition as a promising therapeutic strategy to counter CD8^+^ T cell–induced endothelial death in CM.

## INTRODUCTION

Malaria remains a major global health challenge, causing an estimated 263 million cases and 597,000 deaths in 2023, with about 95% of deaths occurring in the World Health Organization African Region, where children under five accounted for ∼76% of malaria deaths (*1*). Cerebral malaria (CM), the deadliest neurological complication of *Plasmodium falciparum* infection, remains a major cause of malaria-associated death and disability (*1*). CM is clinically defined by unarousable coma with peripheral parasitemia and no alternative cause (*2*, *3*). Despite artesunate-based treatment together with intensive supportive management, case-fatality remains substantial (*4*, *5*), and 25 to 30% of survivors develop long-term neurological sequelae (*2*, *6*). Given the efficacy of artesunate in clearing malaria parasites, central nervous system (CNS) injury is most likely driven by the host immune response and underscores the need for adjunctive treatments that target host-driven pathology, rather than the parasite alone (*2*, *7*). However, the molecular mechanisms linking immune activation to endothelial injury and brain hemorrhage, which are the proximal causes of death, remain incompletely defined (*3*).

CM pathogenesis is driven by the convergence of parasite sequestration on microvessels, host immune responses, and hemostatic dysfunction (*3*, *8*). Postmortem studies reveal congested cerebral microvessels lined with infected red blood cells (RBCs) adherent to the endothelium (*9*, *10*). This is mediated by *Plasmodium*-derived surface proteins binding endothelial receptors, leading to microvascular obstruction and ischemia (*8*). Notably, malaria is frequently accompanied by thrombocytopenia and coagulopathy, including features of disseminated intravascular coagulation, potentially increasing the risk of cerebral microhemorrhages (*11*–*13*). Brain imaging and autopsy studies frequently show petechial hemorrhages, perfusion abnormalities, and brain swelling in children with human CM (*14*–*17*). Concurrently, infected RBCs and pathogen antigens induce high levels of proinflammatory cytokines, which have been implicated in endothelial tight-junction disruption, blood-brain barrier (BBB) permeability, and vasogenic edema (*18*, *19*). Together, BBB leakage, brain swelling, and multifocal hemorrhages are hallmark features of human CM. Experimental models recapitulate aspects of this vascular pathology but differ in parasite cytoadhesion and the relative contributions of immune mechanisms (*3*).

Among host factors, in mouse CM, CD8^+^ T cells have emerged as central mediators of neurovascular damage (*20*–*22*). In the *Plasmodium berghei* ANKA (PbA) mouse model, depletion of CD8^+^ T cells or loss of their cytotoxic effector functions prevents CM without reducing parasite burden (*20*, *21*). Perforin-deficient mice are resistant to CM, yet the adoptive transfer of wild-type CD8^+^ T cells restores the fatal phenotype (*21*). Brain endothelial cells up-regulate major histocompatibility complex (MHC) class I and cross-present parasite antigens, enabling antigen-specific CD8^+^ T cells to arrest on cerebral microvessels and induce localized vascular leakage (*23*–*27*). In fatal pediatric human CM, effector CD8^+^ T cells associate with the cerebral vasculature, and their abundance correlates with CM status and burden of parasite sequestration (*28*, *29*). The endothelial death pathways engaged in mouse CM and their relationship to human disease remain unclear (*3*).

Cytotoxic T lymphocytes (CTLs) typically induce target-cell death through perforin and granzyme release (*30*). However, during severe inflammation, multiple cell death pathways, including apoptosis and necroptosis, can be activated in parallel or in intertwined cascades (*31*). Such pathway overlap has been described in response to intense inflammatory stimuli (*31*–*33*). These pathways can be linked by shared upstream inflammatory and cell death signaling molecules (*32*, *33*). A central regulator of inflammatory cell death signaling is the receptor interacting serine/threonine kinase 1 (RIPK1), which functions as a checkpoint in death receptor signaling (*34*). Under homeostatic conditions, RIPK1 primarily supports prosurvival signaling (*34*). However, when death receptors are engaged in the presence of interferon signaling, RIPK1 recruits RIPK3 to form the necroptotic complex, culminating in mixed lineage kinase domain-like protein (MLKL) phosphorylation and membrane rupture (*35*). Moreover, RIPK1 can interact with inflammasome pathways and apoptotic cascades, especially when multiple death triggers are present (*33*, *36*). Because of its integrator role, RIPK1 has been identified as a potential therapeutic target for diseases characterized by uncontrolled inflammation and cell death (*35*). RIPK1 inhibitors are in development for inflammatory and neurodegenerative conditions (*37*–*39*), yet it remains unknown whether RIPK1-mediated cell death contributes to CM development. Here, we identify a CD8^+^ T cell–dependent, RIPK1-centered regulated cell death program in brain endothelial cells in mouse CM, test its pharmacological tractability, evaluate endothelial RIPK1 activation in human CM tissue, and assess the genetic association of RIPK1 with severe malaria.

## RESULTS

### Regionally selective vulnerabilities to microhemorrhage in human CM

To establish a human reference for the regional distribution of vascular injury during CM, we analyzed susceptibility-weighted imaging (SWI) scans from an Indian cohort comprising 10 patients with CM and five healthy adult controls (*14*–*17*). The human CM cohort included both children and adults (median age, 23 years; range, 5 to 47) presenting with profound coma (median GCS = 5.5; table S1). The healthy control group consisted of volunteers aged 20 to 69 years.

Microbleeds were identified in a subset of 10 patients with CM (table S1) drawn from the larger Rourkela magnetic resonance imaging (MRI) cohort (*40*), appearing as diffuse punctate hypointensities on SWI sequences, measuring 1 to 5 mm in diameter (Fig. 1, A and B, and movie S1). No microhemorrhages were observed in the healthy controls. Quantitative spatial mapping revealed a highly selective anatomical distribution (Fig. 1C). The lesion burden was most profound in the corpus callosum and juxtacortical white matter, matching the U-fiber distribution observed in the schematic reconstruction (Fig. 1B). In contrast, the cortex and deep gray matter showed no significant accumulation of microbleeds compared to controls. In this CM cohort, microhemorrhage burden was concentrated in the corpus callosum and juxtacortical white matter and was not increased in the cortex or deep gray matter relative to healthy controls.

**Fig. 1.**
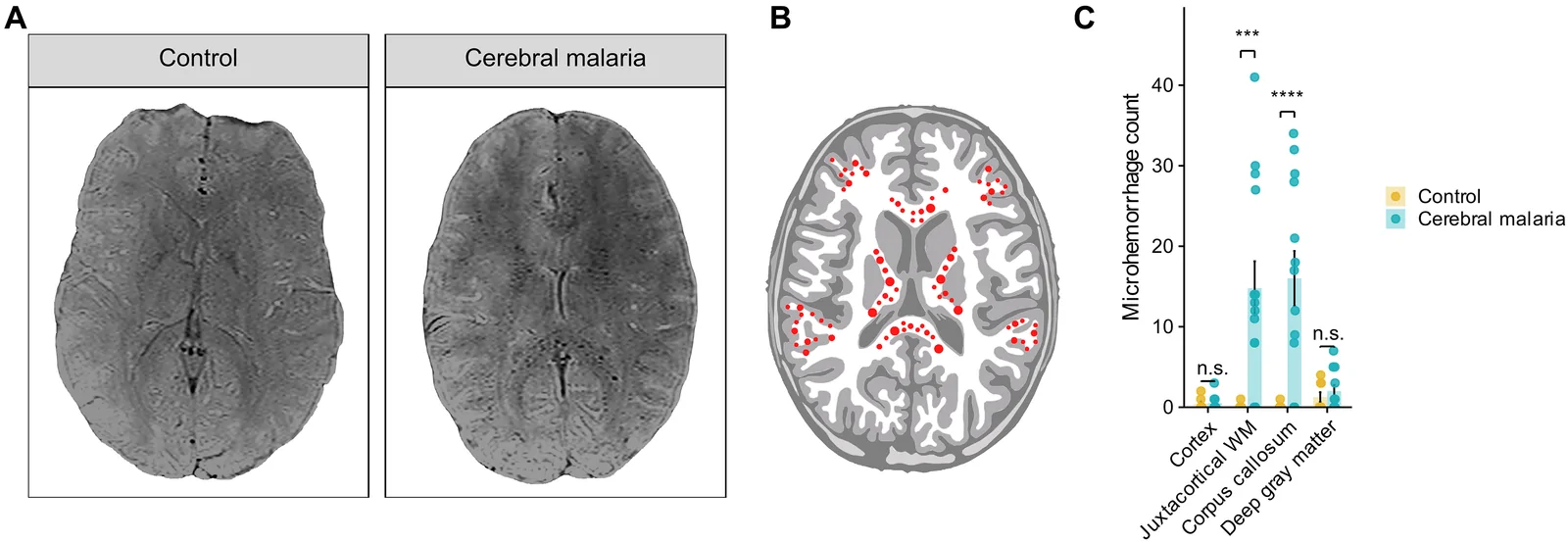
White matter–predominant microhemorrhages in human CM. (**A**) Representative axial SWI sequences from a control participant (left) and a pediatric patient with CM and with microhemorrhages (right). Punctate hypointense foci consistent with microbleeds appear diffusely throughout the juxtacortical white matter and corpus callosum. (**B**) Schematic representation of the microhemorrhage distribution pattern. Red dots indicate the characteristic distribution. (**C**) Quantitative analysis of microhemorrhage count across anatomical regions (*n* = 5 controls; *n =* 10 patients with CM). Scatter dot plot comparing the number of microhemorrhages in controls versus patients with CM across the five indicated brain regions. Horizontal bars represent group means. Counts reflect the total number of microhemorrhages per patient per region, summed across all axial SWI slices. Unpaired two-tailed *t* tests were used. ***P < 0.001, ****P < 0.0001; n.s., not significant.

### Spatially resolved hemorrhage patterns in mouse CM

To determine whether similar regional hemorrhage vulnerabilities exist in the PbA experimental model, we used correlative microscopy across biological scales in C57BL/6J mice.

Infection was initiated with intraperitoneal injection of infected RBCs, with analysis performed on day 6 postinfection (p.i.), coinciding with the onset of neurological symptoms (Fig. 2A). Plasma neurofilament light chain (NfL), a sensitive marker of neuroaxonal injury (*41*), was significantly elevated in PbA-infected mice compared to mock-infected controls (Fig. 2B), confirming acute neuroaxonal damage.

**Fig. 2.**
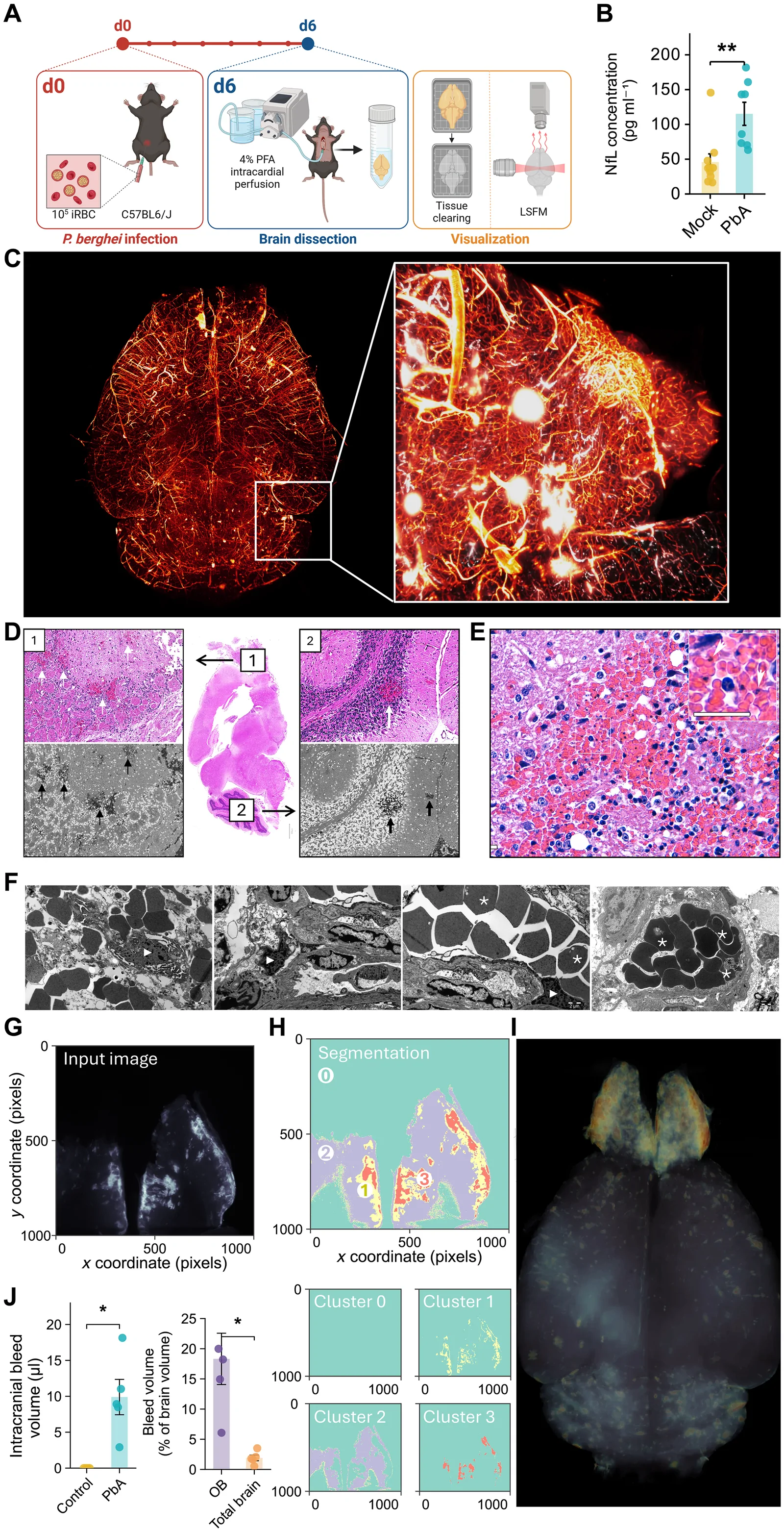
Region-specific hemorrhagic pathology in mouse CM. (**A**) Experimental timeline. C57BL/6J mice were infected with PbA-infected RBCs [1 × 10^5^, intraperitoneally (ip)] and analyzed at day 6. (**B**) Plasma NfL at day 6 (*n* = 8 PbA; *n* = 10 controls). (**C**) Three-dimensional LSFM reconstruction of a cleared PbA-infected brain (tomato lectin). Inset: Hemorrhagic foci. Scale bars, 2 mm (overview) and 500 μm (inset). (**D**) Hematoxylin and eosin (H&E)–stained sagittal section with magnified regions showing perivascular ring hemorrhages (arrows). Scale bars, 2 mm (overview) and 100 μm (insets). (**E**) High-magnification H&E staining showing extravasated RBCs and inflammatory infiltrates. Scale bar, 50 μm. (**F**) Transmission electron microscopy of cerebral microvasculature. The second and third images show partially overlapping views of the same microvascular region. Arrowheads, degenerating endothelial cells; asterisks, sequestered parasitized RBCs. Scale bars, 2 μm. (**G**) Autofluorescence input image for hemorrhage segmentation. (**H**) Automated segmentation output with k-means clustering into four hemorrhagic classes. (**I**) Dorsal photograph of a PbA-infected brain showing macroscopic hemorrhage. (**J**) Left: Total bleed volume (*n* = 5 PbA; *n* = 4 control). Right: Bleed volume as percentage of brain volume for olfactory bulbs (OB) versus total brain. Unpaired two-tailed *t* test; *P < 0.05. Data are means ± SEM. Figure 2A was created in BioRender. Hadjilaou, A. (2026) https://BioRender.com/cnv336y.

Following intracardiac perfusion with tomato lectin to label the vasculature, whole brains were optically cleared and imaged by light-sheet fluorescence microscopy (LSFM) to map hemorrhage-associated signal across the brain at mesoscale resolution (Fig. 2C and movie S2). Whole-brain projections from the complete control and PbA-infected cohorts, together with representative higher-resolution images, are shown in fig. S1. With lateral sampling of 5.26 to 6.55 μm and an axial step of 5 μm, this approach does not resolve endothelial junctions or directly measure microvascular permeability.

To validate the nature of the observed vascular lesions, we performed histopathological analysis on matched brain sections. Hematoxylin and eosin staining confirmed ring hemorrhages surrounding small vessels, particularly in white matter tracts (Fig. 2, D and E, and movie S3). Correlative electron microscopy showed microvascular damage, including endothelial injury, infected RBC adherent to the vessel wall, and pericyte degeneration with basement membrane abnormalities (Fig. 2F). Together with the histological ring hemorrhages, these ultrastructural findings support that the LSFM-detected lesions represent actual vascular rupture.

We next quantified these hemorrhagic lesions across the entire cohort using an automated segmentation pipeline. Clustering of olfactory bulb images identified four distinct hemorrhagic phenotypes: perivascular microbleeds, diffuse hemorrhagic zones, punctate hemorrhages, and areas of confluent bleeding (Fig. 2G). These clusters were used to train a neural-network classifier, enabling high-throughput quantification of hemorrhage distribution (Fig. 2, H and I, and movie S4). Volumetric quantification revealed significantly greater intracranial hemorrhage volume in infected mice than in controls (Fig. 2J). When normalized to brain volume, hemorrhagic burden was greater in the olfactory bulbs than in the brain as a whole (Fig. 2J). This olfactory-bulb enrichment aligns with prior observations of edema and damage preferentially affecting the olfactory bulb and brainstem in mouse CM (*42*). Together, these findings show that human and mouse CM exhibit regionally selective hemorrhage, but the affected territories differ between species.

### Systemic coagulopathy in mouse CM

Hemorrhage in mouse CM may arise from a systemic prohemorrhagic state, focal vascular injury, or both. We first evaluated the systemic axis by assessing coagulation status across stages of mouse CM severity.

Complete blood counts on day 6 p.i. revealed severe thrombocytopenia, with platelet counts dropping by more than 90%, consistent with a failure of primary hemostasis. Concurrently, leukocytosis was observed, including marked elevations in neutrophils, monocytes, and eosinophils. In contrast, lymphocyte counts declined (Fig. 3A). Correlation analysis revealed coordinated hematologic dysregulation in PbA-infected mice (Fig. 3B). Notably, lymphocyte counts and the disease severity score were not significantly correlated with other hematological parameters. To evaluate functional coagulation capacity, we performed calibrated automated thrombography (*43*). While baseline endogenous thrombin potential (ETP) remained similar between groups, activation via the intrinsic (kaolin triggered) and extrinsic pathway (tissue factor initiated) revealed significantly impaired thrombin generation in PbA-infected mice (Fig. 3, C and D). ETP was reduced by ∼30% in both triggers, whereas the thrombograms showed short lag times and rapid peak formation in both PbA-infected and noninfected control mice.

**Fig. 3.**
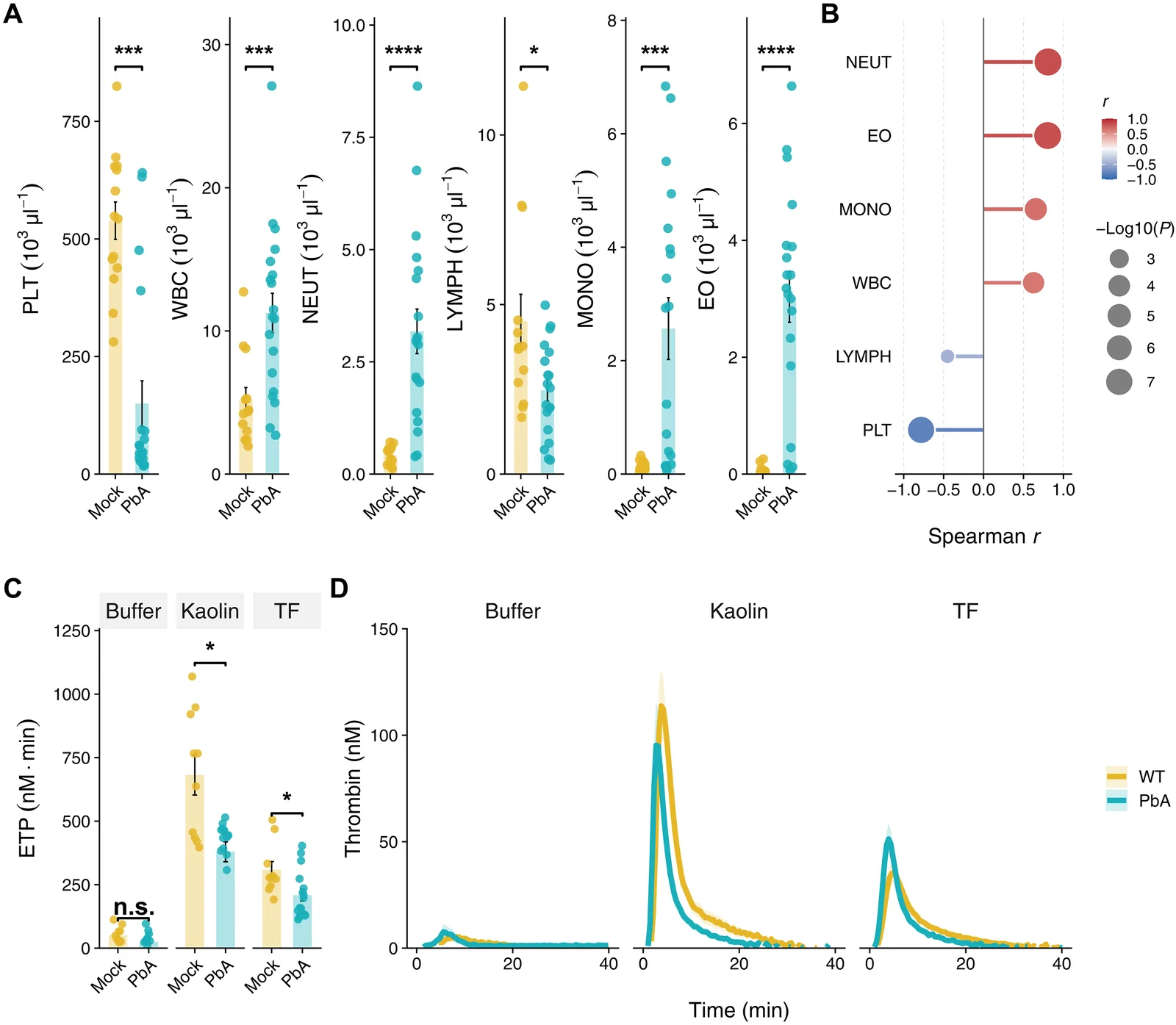
Impairment of primary and secondary hemostasis in experimental CM. (**A**) Complete blood count analysis at day 6 postinfection (*n* = 19 per group). PLT, platelets; WBC, white blood cells; NEUT, neutrophils; LYMPH, lymphocytes; MONO, monocytes; EO, eosinophils. (**B**) Lollipop plot showing correlation between hematological parameters and disease severity score in PbA-infected mice. Point position on the *x* axis indicates the correlation coefficient (*r*); point size is proportional to −log_10_(*P* value); color reflects correlation direction and magnitude (blue, negative; red, positive). Only statistically significant correlations are shown (*P* < 0.05). (**C**) ETP was measured by calibrated automated thrombography under baseline conditions (buffer), intrinsic pathway activation (kaolin), and extrinsic pathway activation [tissue factor (TF)]. (**D**) Mean thrombin generation curves in WT and PbA-infected mice. Data are presented as means ± SEM, with individual data points. For (A) and (C), Mann-Whitney *U* test was used. For (B), Spearman rank correlation. *P < 0.05, ***P < 0.001, ****P < 0.0001.

These findings demonstrate concurrent defects in primary (platelet-mediated) and secondary (coagulation-mediated) hemostasis in mouse CM. This systemic vulnerability may set the stage for bleeding but does not explain its regional topography, which instead appears to reflect sites of focal endothelial injury.

### Ribosome profiling identifies CD8^+^ T cell–dependent endothelial cell death programs

Prior work showed that endothelial MHC class I is required for CD8^+^ T cell vascular engagement, targeted cell death, and regional BBB disruption in mouse CM (*25*, *27*, *44*). To define the molecular drivers associated with this endothelial injury, we sought to characterize the translational signature of brain endothelial cells during mouse CM.

To this end, we performed endothelium-specific ribosome profiling using translating ribosome affinity purification (TRAP) in *Tie2-L10a-eGFP* transgenic mice (Fig. 4A) (*45*). Immunofluorescence confirmed endothelial specificity, with green fluorescent protein (GFP) expression restricted to CD31^+^ endothelial cells and absent expression in NeuN^+^ neurons, Iba-1^+^ microglia, MBP^+^ oligodendrocytes, and glial fibrillary acidic protein–positive astrocytes (fig. S2A).

**Fig. 4.**
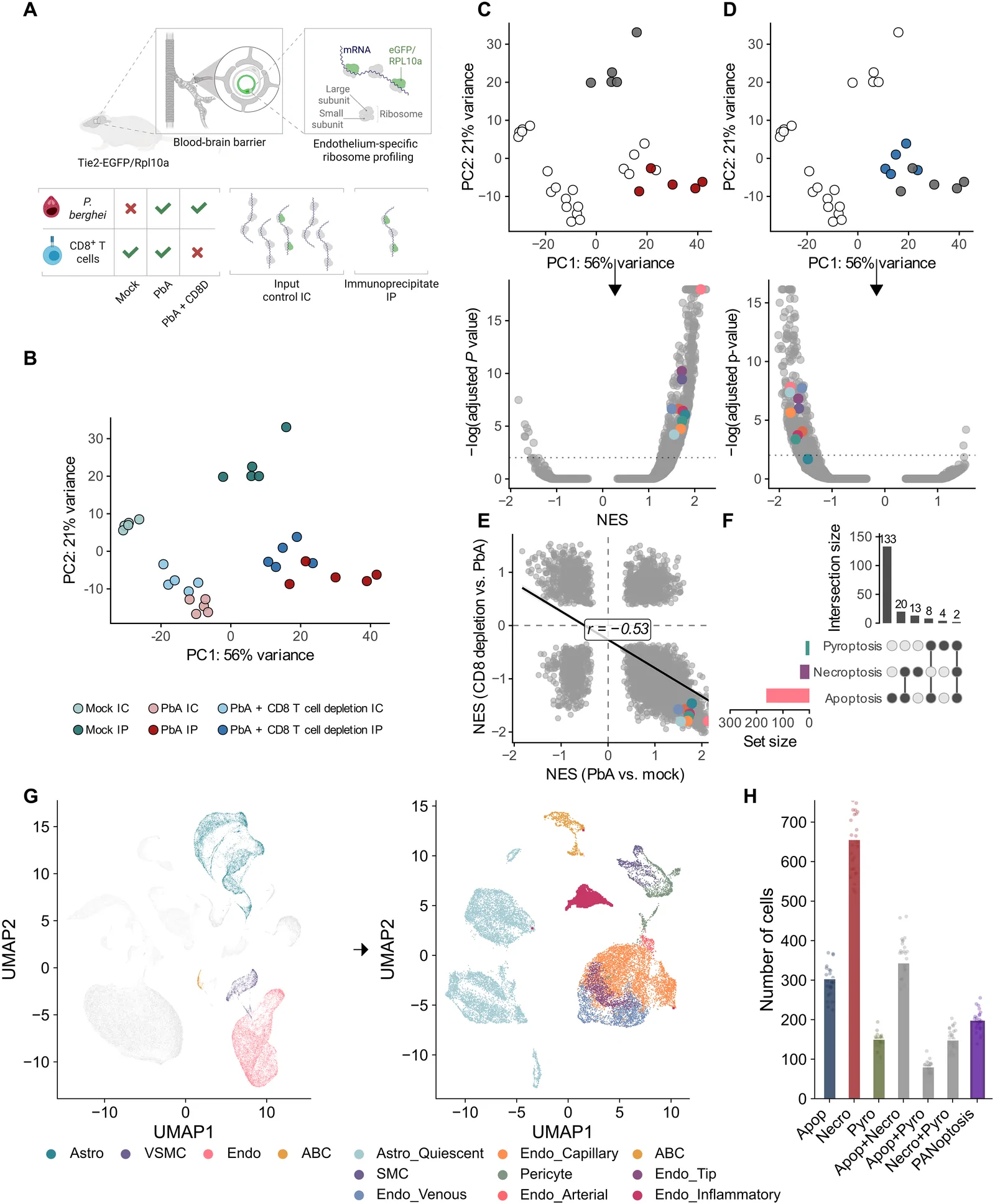
Convergent regulated cell death in brain endothelial cells in mouse CM. (**A**) TRAP-seq schematic. *Tie2-L10a-eGFP* mice were used for endothelial ribosome immunoprecipitation (IP) from mock-infected, PbA-infected, and CD8^+^ T cell–depleted conditions. IC, input control (whole brain). (**B**) PCA of endothelial-enriched IP RNA-seq samples (*n* = 5 per group). Exact-permutation PERMANOVA: pseudo-*F*(2,12) = 24.67, *R*^2^ = 0.804, *P* = 3.17 × 10^−5^; pairwise Holm-adjusted *P* = 0.032 (anti-CD8 versus PbA) and *P* = 0.024 (anti-CD8 versus mock); dispersion *P* = 0.502. (**C**) GSEA of regulated cell death (RCD) pathways in endothelial IP: PbA versus mock. Pathways colored by RCD category: tumor necrosis factor superfamily cytokine production (salmon pink), extrinsic apoptotic signaling pathway (mauve), response to tumor necrosis factor (muted violet), regulation of apoptotic signaling pathway (slate blue), positive regulation of apoptotic signaling pathway (pale blue), extrinsic apoptotic signaling pathway via death domain receptors (rasberry pink), programmed necrotic cell death (teal blue), endothelial cell apoptotic process (orange), granzyme mediated programmed cell death signaling pathway (sea green), and regulation of extrinsic apoptotic signaling pathway (terracotta). (**D**) GSEA comparing PbA + CD8^+^ T cell depletion versus PbA alone; FDR < 0.05. (**E**) Normalized enrichment score (NES) correlation between contrasts (*r* = −0.53). (**F**) UpSet plot of pathway overlap across curated regulated cell death gene sets. (**G**) scRNA-seq reanalysis of Chen *et al.* (*46*): UMAP of neurovascular unit and endothelial subclustering. (**H**) RCD pathway coactivation in inflammatory endothelial cells (AUCell, threshold: mean + 1 SD). Data are means ± SEM. In (C) and (D), fgsea; FDR < 0.05. In (E), Pearson correlation. In (H), AUCell; threshold: mean + 1 SD. Figure 4A was created in BioRender. Hadjilaou, A. (2026) https://BioRender.com/0lsxk3g.

To determine how CD8^+^ T cells, established drivers of mouse CM (*20*–*22*), shape the brain endothelial response, we performed RNA sequencing (RNA-seq) of whole-brain tissue [input controls (IC)] and immunoprecipitated brain endothelial cell–specific ribosomes (IP) under three experimental conditions: mock-infected controls, PbA infection, and PbA infection with CD8^+^ T cell depletion. CD8^+^ T cells were depleted by administration of anti-CD8 antibodies on days 1 and 3 p.i., achieving >95% depletion, as confirmed by flow cytometry, and complete rescue from clinical mouse CM symptoms (fig. S2B).

After sequencing, we validated the specificity of our enrichment using gene set enrichment analysis (GSEA) with cell type–specific marker genes (fig. S3). This confirmed robust enrichment of endothelial cell markers in the IP samples across all conditions, with corresponding depletion of markers for neurons, astrocytes, oligodendrocytes, microglia, and mural cells. Principal components analysis (PCA) revealed clear separation by infection status (Fig. 4B). Exact-permutation permutational multivariate analysis of variance (PERMANOVA) of the endothelial-enriched IP samples (*n* = 5 per group; IC samples excluded) confirmed group separation on PC1 and PC2 scores [pseudo-*F*(2,12) = 24.67, *R*^2^ = 0.804, *P* = 3.17 × 10^−5^], without evidence of unequal dispersion (*P* = 0.502). The anti-CD8 centroid was partially shifted from PbA toward mock, lying 33.7% of the distance from the PbA to the mock centroid [95% confidence interval (CI), 17.8 to 48.0%]; pairwise Holm-adjusted *P* values were 0.032 versus PbA and 0.024 versus mock.

Enrichment analysis showed significant up-regulation of several cell death pathways in brain endothelial cells of PbA-infected mice (Fig. 4C). Apoptosis-related genes showed the strongest enrichment, accompanied by activation of both extrinsic caspase signaling and intrinsic apoptotic pathways. In addition, we observed enrichment of inflammatory cell death markers and RIPK1-mediated necroptosis. Crucially, comparison of IP samples from CD8^+^ T cell–depleted infected mice versus nondepleted infected mice revealed that these death signatures were markedly attenuated in the absence of CD8^+^ T cells (Fig. 4D). Enrichment of apoptosis, caspase activation pathways, and necroptosis was reduced in depleted animals, demonstrating a CD8^+^ T cell–dependent component of the brain endothelial death program without excluding contributions from other immune compartments. Consistent with this, a global comparison of pathway enrichment scores revealed a strong negative correlation between the two contrasts (*r* = −0.53; Fig. 4E), indicating that the same death-related programs up-regulated during PbA infection are reversed upon CD8^+^ T cell depletion. To determine whether these cell death modalities were activated concurrently rather than independently, we cross-referenced the leading-edge genes from significantly enriched pathways against curated gene sets from the Regulated Cell Death database (RCDdb). UpSet analysis revealed substantial overlap across regulated cell death modalities, with a shared core of death-related genes (Fig. 4F).

To assess these programs at single-cell resolution, we reanalyzed published mouse CM single-cell RNA-seq data from noninfected, PbA-infected, and artesunate-treated brains (*46*) (Fig. 4G). After inflammatory module scoring and endothelial reclustering, we defined an analysis-derived Endo_Inflammatory state marked by *Vcam1*, *Ccl3*, and *Ccl4*. Per-replicate abundance was 1.01 ± 0.13% of endothelial cells in noninfected controls, 5.50 ± 5.92% in mouse CM (range, 0.38 to 11.98%), and 9.62 ± 1.27% after artesunate treatment (*n* = 3 mice per condition). The overall difference in abundance was not significant (exact-permutation Kruskal-Wallis test, *H* = 3.20, *P* = 0.254); accordingly, we describe this state as rare in the uninfected brain and variably expanded in disease rather than as infection-specific. Within this state, individual cells showed coactivation of overlapping regulated cell death programs (Fig. 4H). Together, these analyses identify a CD8^+^ T cell–dependent endothelial transcriptional program enriched for cell death–related genes in mouse CM.

### Brain-infiltrating CD8^+^ T cells exhibit a cytotoxic signature in mouse CM

To define the molecular programs underlying CD8^+^ T cell–mediated brain endothelial cell injury in mouse CM, we used a dual transcriptomic approach (Fig. 5A). We performed RNA-seq on flow cytometry–sorted antigen-experienced (CD44^hi^) CD8^+^ T cells isolated from the brain tissue of infected mice, comparing them to CD44^hi^ CD8^+^ T cells from the spleens of both infected and uninfected control mice.

**Fig. 5.**
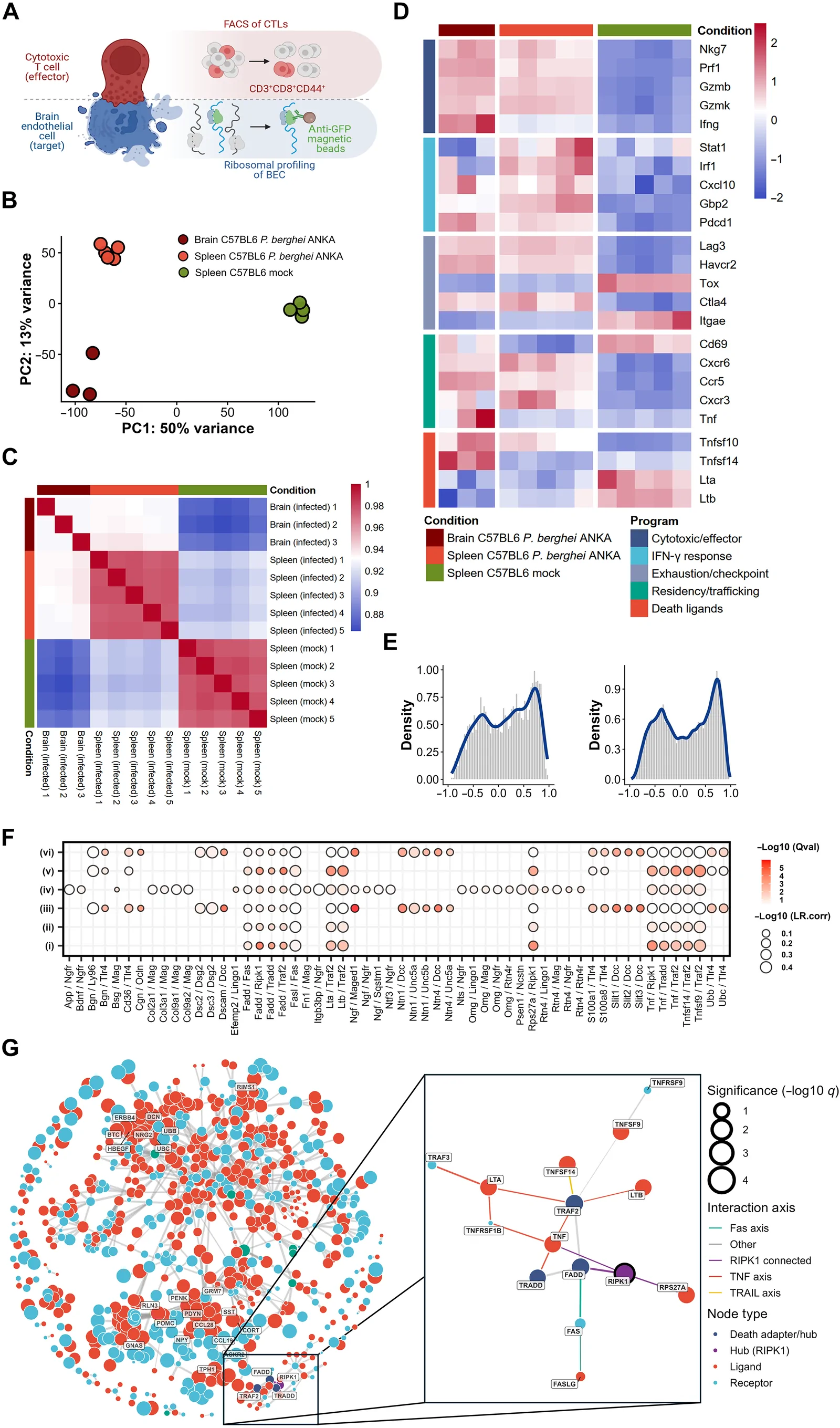
RIPK1 as a convergence point of CD8^+^ T cell–endothelial death signaling. (**A**) Schematic: brain-infiltrating CD8^+^ T cells (CD3^+^CD8^+^CD44^+^) were sorted from infected mice; brain endothelial cells (BEC) underwent TRAP-seq to construct the immunocerebrovascular interactome. (**B**) PCA of CD8^+^ T cell transcriptomes: brain (*n* = 3), spleen PbA-infected (*n* = 5), spleen mock (*n* = 5). (**C**) Sample-to-sample Spearman correlation matrix. (**D**) Heatmap of CD8+ functional gene modules (row-wise *z*-scores; visualization only). Gene-level differential expression between brain-infiltrating and infected-spleen CD8^+^ T cells was tested using DESeq2 with Benjamini-Hochberg correction across 20,077 genes; per-gene log2 fold change, 95% CIs, Wald *z* statistics, *P* values, and FDR-adjusted *q* values are reported in table S3. (**E**) BulkSignalR parameter learning: LR and RT Spearman correlation nulls. (**F**) Ligand-receptor bubble plot across death pathways: (i) RIPK1-mediated regulated necrosis, (ii) regulated necrosis, (iii) programmed cell death, (iv) death receptor signaling, (v) caspase activation via death receptors, (vi) apoptosis. Circle color: −log10(q); circle size: L-R correlation. (**G**) Global L-R network (left) and RIPK1-centered subnetwork (right). In (B), DESeq2 VST-transformed counts. In (D), *z*-scores clipped to [−2, 2]. In (F), BulkSignalR with Spearman correlation-based null; *q* ≤ 0.001. In (G), edges represent significant interactions. Figure 5A was created in BioRender. Hadjilaou, A. (2026) https://BioRender.com/dgv1ocg.

PCA revealed a fundamental divergence between the transcriptional landscapes of brain- and spleen-derived CD8^+^ T cells (Fig. 5B). Brain-infiltrating cells from infected mice formed a cluster distinct from both infected and control splenic populations along PC1, which accounted for 50% of the variance. Spearman correlation analysis revealed a similar pattern, with high within-group reproducibility and low correlations among tissue compartments (Fig. 5C). These data indicate that brain-infiltrating CD8^+^ T cells acquire a transcriptional program distinct from the systemic immune response.

Formal unpaired differential expression of brain-infiltrating versus splenic CD44^hi^CD8^+^ T cells from PbA-infected mice revealed a selective rather than uniform transcriptional program (Fig. 5D). *Tnf* [log_2_ fold change = 1.45, false discovery rate (FDR) = 3.9 × 10^−6^] and *Fasl* (log_2_ fold change = 1.37, FDR = 3.1 × 10^−14^) were enriched in brain-derived cells. *Gzmb*, *Cxcl10*, *Pdcd1*, and *Lag3* did not differ between compartments, whereas *Stat1*, *Irf1*, *Tox*, and *Itgae* were lower in brain-derived cells. Formal differential expression showed selective enrichment of *Tnf* and *Fasl* without broader brain-specific enrichment of canonical exhaustion or checkpoint markers (table S3).

To define the functional consequences of this signature on the BBB, we constructed an immunocerebrovascular interactome by integrating our brain endothelial ribosome profiling data with the CD8^+^ T cell transcriptomes. To ensure robust inference, we empirically estimated null distributions for ligand-receptor and receptor-target gene correlations from the merged transcriptomic dataset. These distributions confirmed that the observed ligand-receptor correlations in our immunocerebrovascular interactome significantly exceeded chance expectation, validating downstream pathway enrichment analysis (Fig. 5E). Using this framework, we identified multiple cell death–related signaling cascades as the most significantly enriched interactions (Fig. 5F). “RIPK1-mediated regulated necrosis” emerged as a top-ranking pathway, accompanied by significant enrichment for “Death Receptor Signaling” and “Apoptosis.” This analysis generated a global interaction network that identified RIPK1 as a convergence point for T cell–endothelial death signaling (Fig. 5G). Specifically, the up-regulation of *Tnf*, *Fasl*, and *Tnfsf10* in brain-infiltrating CD8^+^ T cells linked directly to their cognate receptors (*TNFR1*, *Fas*, and *DR5*) and the downstream RIPK1 hub on endothelial cells.

The integrated analysis identifies death-ligand signaling that converged on a RIPK1-centered endothelial network in mouse CM.

### Cell death signaling pathway activation in mouse and human CM

To validate the activation of the cell death programs identified in our transcriptomic analyses at the protein level, we quantified the expression of key executioner molecules in the brains of PbA-infected mice compared with uninfected controls. We performed quantitative histological analysis of brain sections stained for cleaved caspase 3 (cCASP3), a marker of apoptosis, and phosphorylated MLKL (pMLKL, S345), the terminal effector of necroptosis.

Examination of distinct brain regions revealed a significant, infection-induced up-regulation of both death markers (Fig. 6, A and B, and fig. S4). The necroptotic marker pMLKL showed robust induction, with significant increases in the brain cortex, brainstem, and olfactory bulbs (Fig. 6A). Concurrently, the levels of the apoptotic executioner protease cCASP3 were significantly elevated in the brainstem and olfactory bulbs of PbA-infected animals compared to controls (Fig. 6B). Attempts to detect pRIPK1(S166) in mouse brain sections using published protocols did not yield a specific signal; therefore, endothelial pRIPK1 localization was assessed only in human tissue.

**Fig. 6.**
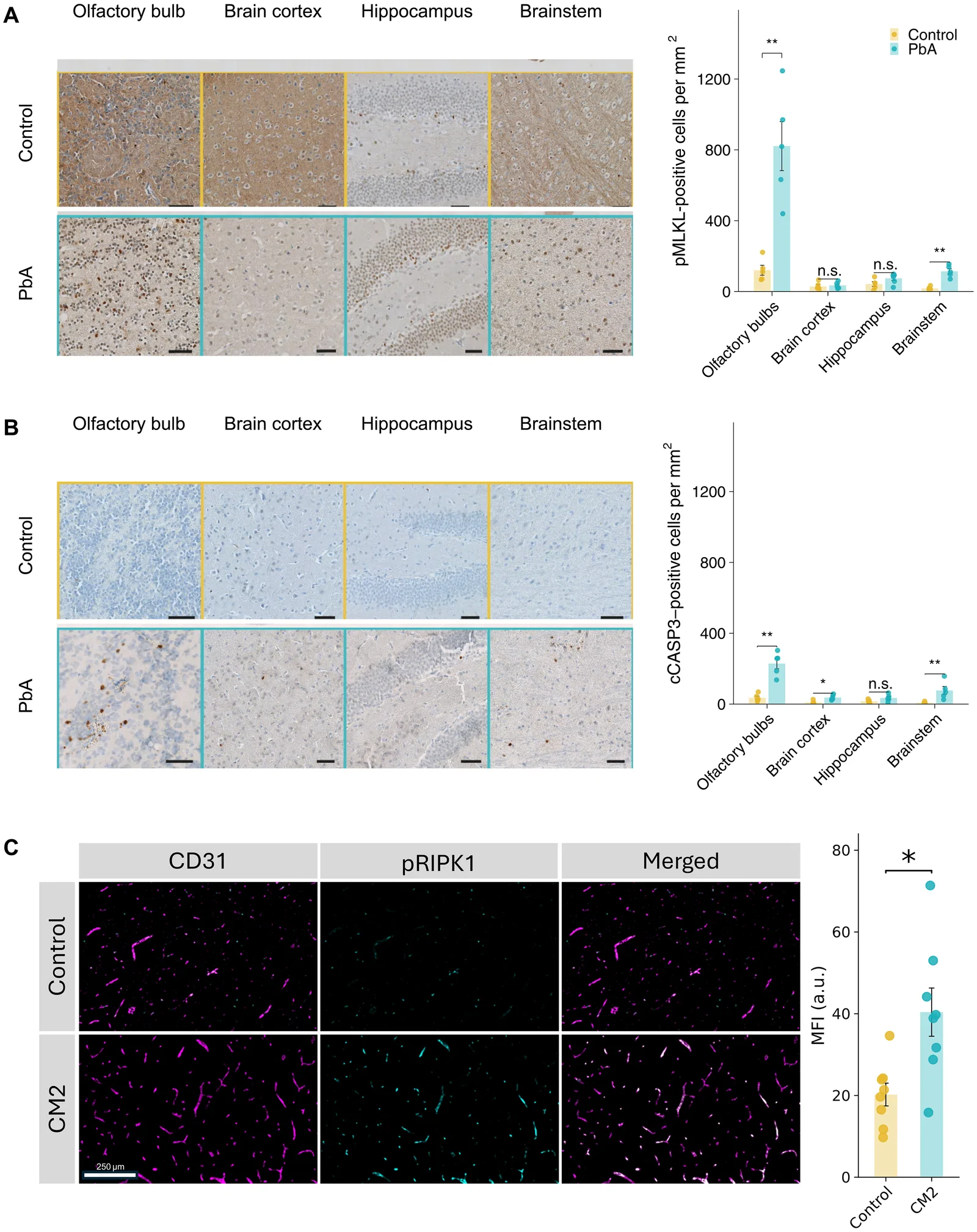
Cell-death pathway activation in mouse and human CM. (**A** and **B**) Representative DAB-based chromogenic immunohistochemistry images (left) and corresponding quantification (right) of (A) pMLKL (S345) and (B) cCASP3-positive cells in the indicated brain regions from control and PbA-infected mice (*n* = 5 to 8 per group). Data are shown as positive cells per mm^2^. Scale bars, 50 μm; corresponding overview images of analyzed areas in (A) and (B) are shown in fig. S4. (**C**) Immunofluorescence of postmortem brain tissue from pediatric human CM fatalities (CM2, CM pathological phenotype 2) and fatal nonmalaria encephalopathy controls stained for CD31 (magenta) and pRIPK1 (cyan). Scale bar, 250 μm. Right: pRIPK1 mean fluorescence intensity within CD31^+^ vessels; each point represents a per-patient mean (*n* = 8 CM; *n* = 8 controls). In (A) and (B), bars show means ± SEM with individual data points. Mann-Whitney *U* test; *P < 0.05, **P < 0.01.

To establish the translational relevance of these findings, we examined postmortem brain tissue from pediatric human CM fatalities and nonmalaria controls from Blantyre, Malawi (clinical characteristics summarized in table S2). Immunofluorescence analysis revealed that pRIPK1 (S166) immunoreactivity was strongly detected in the cerebral blood vessels of patients with CM, where it colocalized extensively with the endothelial marker CD31 (Fig. 6C). Quantitative analysis confirmed a significantly increased pRIPK1 staining intensity within the endothelium of human CM cases compared with controls.

### Polygenic association of regulated cell death genes with human malaria severity

To test whether cell death pathways contribute to CM susceptibility independently of our experimental findings, we curated a high-confidence gene set (622 genes) compiled from the RCDdb (*47*) and performed subphenotype-stratified GWAS across eight MalariaGEN cohorts (*48*). CM showed enrichment of regulated cell death pathways above the genome-wide background in six of eight cohorts (fig. S5 and table S4), but individual analyses were underpowered because of limited case numbers (33 to 848 per subphenotype). As the genetic architecture of severe malaria is partly shared across subtypes (*48*), we leveraged the composite severe malaria phenotype, 6341 cases and 5992 controls across Gambia, Kenya, and Malawi (Fig. 7A), to maximize power for pathway-level detection. While the genome-wide background showed expected calibration across all cohorts (λ_GC_ = 1.08 to 1.17), variants mapping to regulated cell death loci exhibited pronounced signal inflation (λ_GC_ = 1.60 to 2.12; Fig. 7B). This pathway-specific enrichment yielded a consistent inflation ratio (1.48 to 1.81) across geographically distinct populations (Fig. 7C). Permutation testing confirmed significant enrichment in the Gambian discovery cohort (*P* = 1 × 10^-4^), which replicated in Malawi and Kenya cohorts. The genetic architecture displayed a directional bias with risk alleles predominating in Gambia (74%) and Kenya (61%), whereas Malawi showed a balanced risk-protective profile (Fig. 7D).

**Fig. 7.**
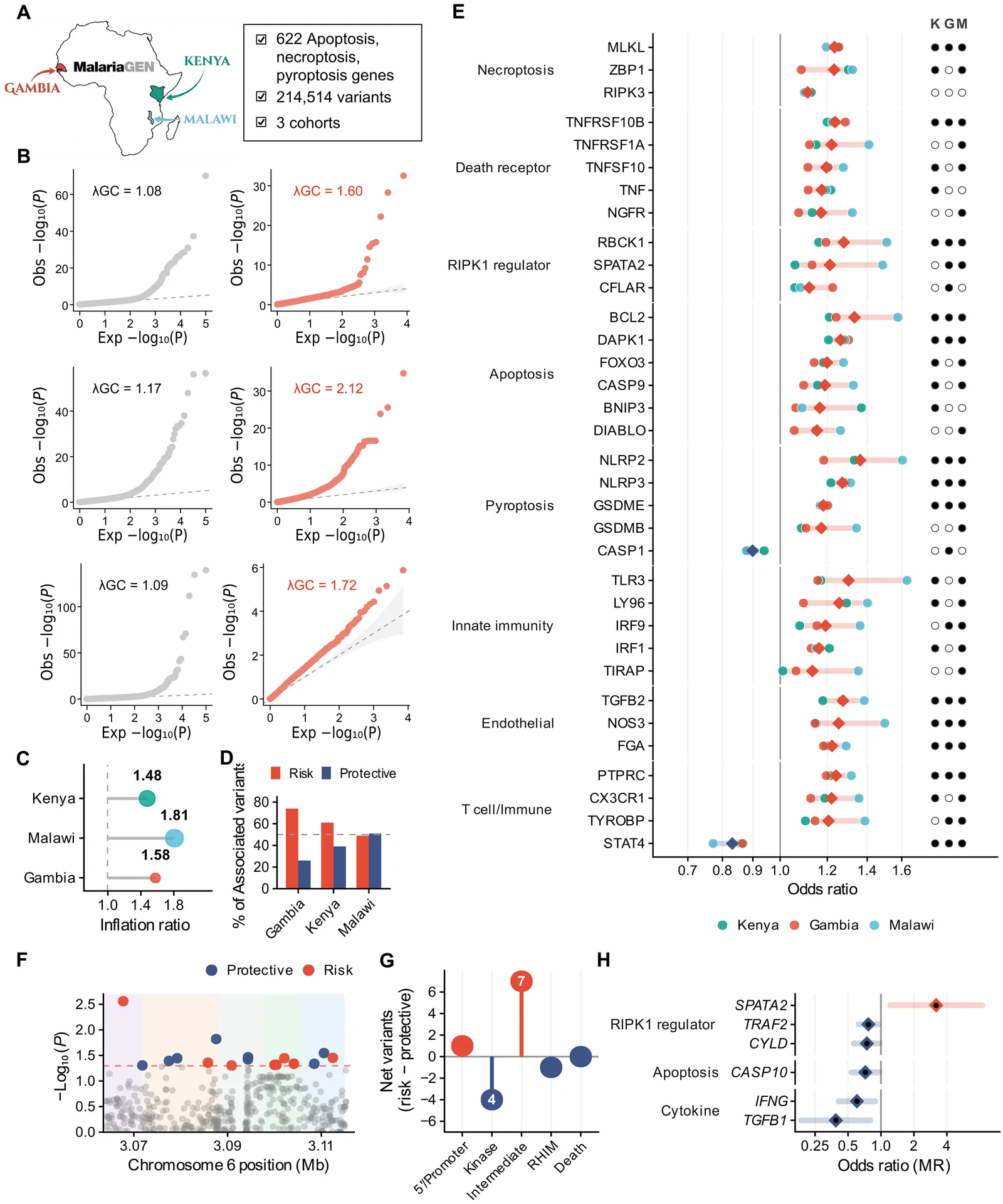
Association of regulated cell death genes with severe human malaria in African populations. (**A**) Study design: 622 regulated cell death genes (214,514 variants) analyzed across Gambia (*n* = 5593), Kenya (*n* = 3652), and Malawi (*n* = 3088); 6341 cases and 5992 controls. (**B**) QQ plots of background (gray) versus regulated cell death pathway variants (red) per cohort. λGC indicated; shaded: 95% CI. (**C**) Cross-cohort pathway-to-background inflation ratios. (**D**) Directional bias of nominally significant variants (*P* < 0.05): risk (red) versus protective (blue). (**E**) Gene-level forest plot by functional category. Filled circles, *P* < 0.05; open, not significant. K, Kenya; G, Gambia; M, Malawi. (**F**) Regional association plot of the *RIPK1* locus (chr 6); colored by effect direction. (**G**) Net variant direction by RIPK1 protein domain. (**H**) Mendelian randomization: OR per 1-SD increase in genetically predicted expression [eQTLGen eQTLs, inverse-variance weighted (IVW) method]. OR < 1: protective; OR > 1: risk. Enrichment assessed by single nucleotide polymorphism–label permutation (10,000 permutations; *P* = 1 × 10^−4^, Gambia). In (F), LD from 1000 Genomes AFR panel (GRCh38). Sensitivity: MR-Egger and Cochran’s Q. In (E), logistic regression (PLINK 2.0). In (H), IVW Mendelian randomization; *P* < 5 × 10^−8^ instrument threshold.

Deconvolution of this signal resolved associations within the RIPK1 regulatory network and downstream executioners (Fig. 7E). We observed replicated risk associations with *RBCK1* (HOIL-1), a component of the linear ubiquitin chain assembly complex (LUBAC), and *CASP8*. Effectors of lytic cell death, including the necroptotic mediator *MLKL* and the lytic cell death effector *GSDME*, also displayed replicated associations. Associations were further observed in upstream sensors [Toll-like receptor 3 (*TLR3*) and *ZBP1*] and death receptors (*TNFRSF10B*).

Fine mapping of the *RIPK1* locus identified a domain-specific distribution of associations (Fig. 7F). The lead promoter variant (rs34610901) was associated with increased risk. Conversely, intronic variants within the kinase-encoding region showed net protective effects (Fig. 7G). A predicted deleterious missense variant in the upstream initiator *ZBP1* (rs41275648) was associated with protection.

Mendelian randomization provided genetically informed estimates for ubiquitin-signaling regulators with known roles in RIPK1 regulation (Fig. 7H). Genetically predicted expression of *SPATA2*, a scaffold that recruits deubiquitinases within ubiquitin-dependent signaling complexes, including RIPK1-containing complexes, was associated with increased risk. In contrast, predicted up-regulation of tumor necrosis factor (TNF) receptor–associated factor 2, a broader ubiquitin-signaling regulator that can ubiquitinate RIPK1 (*49*), was protective. Protective signals were also observed for the apoptotic initiator *CASP10*. These genetic analyses demonstrate enrichment of regulated cell death loci and identify associations within the RIPK1 network in human severe malaria.

### Targeting RIPK1 attenuates CD8^+^ T cell–driven endothelial injury in CM

Having identified RIPK1 as a convergence node for multiple death ligand-receptor interactions between CD8^+^ T cells and brain endothelial cells, we functionally tested this prediction. We established an in vitro assay to mechanistically dissect endothelial cell death following antigen-specific CTL engagement. Primary mouse brain endothelial cells were pulsed with ovalbumin (OVA) and cocultured with flow cytometry–sorted, preactivated splenic effector CD8^+^ T cells isolated from OT-I TCR transgenic mice on day 6 after infection with OVA-expressing PbA (PbTG) (*50*) (Fig. 8A). OVA pulsing models endothelial cross-presentation of parasite antigen, a process that requires endothelial MHC class I for CD8^+^ T cell vascular engagement in vivo (*23*–*25*, *27*). Treatment with either the RIPK1 inhibitor GNE684 or the pan-caspase inhibitor zVAD alone conferred partial protection to endothelial cells, indicating that neither RIPK1 nor caspase signaling accounts for the full extent of antigen-specific CTL-mediated killing. Combined blockade of RIPK1 and caspases restored endothelial viability to levels comparable to unchallenged endothelial-cell controls (Fig. 8B).

**Fig. 8.**
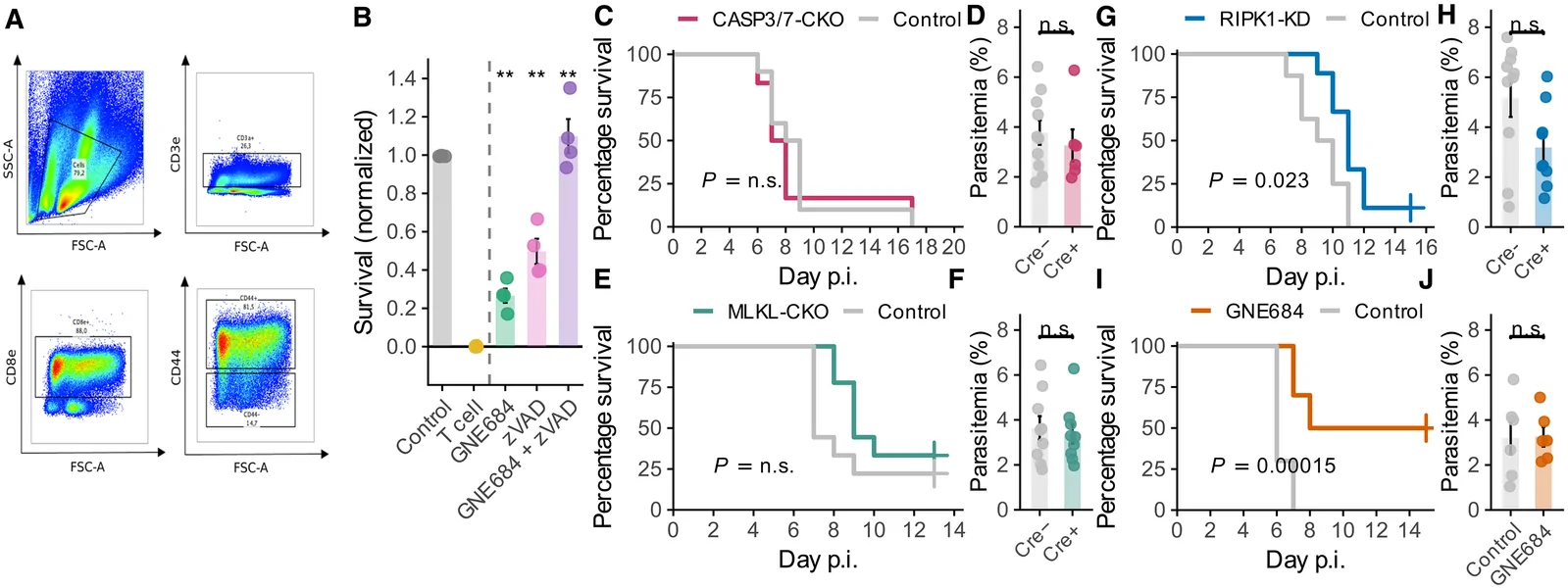
RIPK1- and caspase-dependent endothelial cell death in vitro and mouse CM. (**A**) Flow cytometry gating of OT-I effector CD8^+^ T cells isolated from OT-I transgenic mice at day 6 after infection with OVA-expressing PbA. (**B**) OVA-pulsed endothelial cell survival after coculture with activated OT-I CD8^+^ T cells and treatment with zVAD (pan-caspase inhibitor), GNE684 (RIPK1 inhibitor), or both (*n* = 4 per group). (**C**) Survival of CASP3/7-CKO mice (*Casp3^fl/fl^*;*Casp7^fl/fl^*;Slco1c1-CreER^T2^; *n* = 6) versus controls (*Casp3^fl/fl^*;*Casp7^fl/fl^*, *n* = 10) after PbA infection. (**D**) Parasitemia at day 6 in CASP3/7-CKO and control mice. (**E**) Survival of MLKL-CKO mice (*Mlkl^fl/fl^*;Slco1c1-CreER^T2^; *n* = 9) versus controls (*Mlkl^fl/fl^*; *n* = 9). (**F**) Parasitemia at day 6 in MLKL-CKO and control mice. (**G**) Survival of RIPK1-KD mice (*Ripk1^D138N/fl^*; Slco1c1-CreER^T2^; *n* = 9) versus controls (*Ripk1^D138N/fl^*; *n* = 8). (**H**) Parasitemia at day 6 in RIPK1-KD (*n* = 9) and control mice (*n* = 10). (**I**) Survival of GNE684-treated (20 mg kg^−1^, oral, b.i.d., from day 1 p.i.) versus vehicle-treated control mice (*n* = 10 per group). (**J**) Parasitemia at day 6 in GNE684-treated and vehicle-treated control mice (*n* = 6 per group). In (B), paired *t* test. In (C), (E), (G), and (I), log-rank test. In (D), (F), (H), and (J), Mann-Whitney *U* test. Survival panels [(C), (E), (G), and (I)] each represent one biologically independent cohort per genotype. **P < 0.01, ***P < 0.001, ****P < 0.0001.

We next asked whether the redundancy observed in vitro reflected dispensability of individual executioners in vivo, or whether protection required disabling the upstream kinase at which these pathways converge. To address this, we used the tamoxifen-inducible Slco1c1-CreER^T2^ line, which predominantly targets CNS endothelial cells rather than the systemic endothelium (*51*). Analysis of tamoxifen-treated Slco1c1-CreER^T2^;*Gt(ROSA)26Sor* reporter mice by β-galactosidase activity detected recombination in the brain vasculature but not in peripheral blood cells, bone marrow cells, or spleen (fig. S6), supporting specificity without detectable recombination in the hematopoietic compartment. After tamoxifen induction, endothelial-specific deletion of *Casp3* and *Casp7* (*Casp3^fl/fl^; Casp7^fl/fl^;* Slco1c1-CreER^T2^*;* designated CASP3/7-CKO) showed no significant change in survival or day 6 parasitemia after PbA infection compared with Cre-negative floxed littermate controls (Fig. 8, C and D). Similarly, endothelial-specific *Mlkl*-deficient mice (*Mlkl^fl/fl^;* Slco1c1-CreER^T2^*;* designated MLKL-CKO) showed no significant change in survival or parasitemia compared with Cre-negative floxed littermate controls (Fig. 8, E and F). These results indicate that endothelial loss of the apoptotic executioner caspases 3 and 7, or of the necroptotic executioner MLKL alone, is insufficient to protect from PbA-induced disease.

We therefore evaluated whether inactivating the upstream kinase that licenses coordinated death-pathway activation would alter disease outcome. To this end, we infected mice carrying a kinase-dead *Ripk1^D138N^* allele and a floxed *Ripk1^fl^* allele crossed with the tamoxifen-inducible Slco1c1-CreER^T2^ driver. This strategy generates mice (*Ripk1^D138N/fl^;* Slco1c1-CreER^T2^) in which recombined brain endothelial cells express only the kinase-dead RIPK1 variant after tamoxifen induction (RIPK1-KD). These animals were compared with Cre-negative littermate controls that retain functional RIPK1 expression. Following PbA infection, genetic compromise of RIPK1 kinase activity in the brain endothelium significantly prolonged survival without changing parasitemia (Fig. 8, G and H).

Last, we tested systemic pharmacological RIPK1 inhibition in a separate cohort. GNE684 conferred a survival advantage in PbA-infected wild-type mice (Fig. 8I) without altering parasitemia (Fig. 8J). Together, these experiments show that endothelial RIPK1 kinase activity contributes to mouse CM and that systemic RIPK1 inhibition prolongs survival.

## DISCUSSION

CM remains associated with substantial mortality despite effective antimalarial therapy, underscoring the need for adjunctive strategies that target host pathology (*5*, *15*). Here, we identify RIPK1 as a regulator of CD8^+^ T cell–associated brain endothelial injury in mouse CM. The partial protection conferred by endothelial RIPK1 kinase inactivation indicates that this pathway contributes to, but does not fully account for, lethal vascular pathology.

CD8^+^ T cells are established effectors of neurovascular injury in mouse CM (*20*–*22*, *24*, *30*). Loss of perforin or disruption of endothelial MHC class I prevents vascular engagement, targeted endothelial death, regional BBB disruption, and fatal disease despite comparable parasitemia (*21*, *24*, *44*). Brain endothelial cells are not productively infected by PbA; instead, they can acquire parasite material and cross-present parasite-derived peptides on MHC class I, enabling recognition by antigen-specific CD8^+^ T cells (*22*, *23*, *25*, *26*). Our coculture assay therefore models the cytotoxic interaction downstream of endothelial cross-presentation rather than endothelial infection and implicates RIPK1-regulated target-cell death following CTL engagement. In fatal pediatric human CM, effector CD8^+^ T cells associate with the cerebral vasculature and correlate with sequestration burden (*28*, *29*), but our human analysis does not demonstrate a direct spatial association between these cells and pRIPK1-positive endothelium. The causal CD8^+^ T cell–to–RIPK1 mechanism established here is therefore limited to the mouse model.

Our data further indicate that brain endothelial cells show convergent regulated cell death features during CM, consistent with known overlap between inflammatory cell death pathways (*31*, *32*, *52*). Transcriptional analyses showed up-regulation of caspase-dependent apoptosis, components of inflammatory cell death, and necroptosis effectors. Reanalysis of single-cell RNA-seq data confirmed a distinct inflammatory endothelial population in which individual cells coengaged multiple death pathway programs (*46*). These signatures were attenuated after CD8^+^ T cell depletion, identifying a CD8^+^ T cell–dependent component while leaving room for parallel immune mechanisms. Mechanistically, RIPK1 emerged as a convergence node. While RIPK1 is known to regulate cell-fate decisions (*53*–*55*), under inflammatory conditions, RIPK1 kinase activity drives necroptosis and can also interface with apoptotic and inflammasome signaling (*36*, *56*–*58*). Formal comparison with splenic CD44^hi^CD8^+^ T cells supported selective enrichment of *Tnf* and *Fasl* in brain-infiltrating cells rather than a generalized brain-specific exhaustion or checkpoint program (table S3). These death ligands engage endothelial receptor pathways that can intersect with RIPK1-dependent signaling. In inflammatory environments rich in interferons and TNF, RIPK1 can shift from a prosurvival scaffold to a prodeath kinase that drives necroptosis and amplifies apoptotic and inflammasome signaling (*32*, *35*). Our findings, therefore, connect overlapping inflammatory cell death pathways to infectious cerebrovascular disease and identify RIPK1 as a druggable upstream regulator.

However, our in vitro and in vivo experiments suggest that RIPK1 is one component of a multifactorial death response in CM pathogenesis. In coculture, caspase inhibition provided greater endothelial protection than RIPK1 inhibition alone, consistent with a substantial contribution of perforin/granzyme-mediated cytotoxicity (*21*), although full rescue required inhibition of both pathways. In vivo, endothelial-specific loss of RIPK1 kinase activity significantly prolonged survival but did not prevent terminal disease, indicating that RIPK1-dependent endothelial death is contributing to, but does not fully explain, CM pathology. Systemic pan-caspase inhibition fails to prevent mouse CM (*59*), and endothelial-specific deletion of caspase 3 together with caspase 7 or MLKL was similarly ineffective. Systemic pharmacological inhibition of RIPK1 also prolonged survival, but because it was tested in a separate cohort, its efficacy cannot be directly compared with that of endothelial genetic inactivation. The interventions also differ in cellular scope and coverage. GNE684 inhibits RIPK1 systemically, whereas Slco1c1-CreER^T2^-mediated genetic inactivation primarily targets brain endothelium and may be incomplete, leaving a fraction of endothelial cells with functional RIPK1 kinase activity. Such incomplete coverage could attenuate the genetic phenotype. In addition, sustained genetic inactivation of endothelial RIPK1 kinase activity may permit compensatory engagement of RIPK1-independent death pathways, which are less prominent during transient pharmacological inhibition. Systemic RIPK1 inhibition may also dampen inflammatory signaling in multiple cell types, including infiltrating immune cells, thereby limiting feed-forward inflammatory circuits that amplify vascular injury. This interpretation is consistent with evidence that inflammatory cytokine production also contributes to mouse CM pathology (*60*). RIPK1 inhibition may therefore provide broader protection than single-pathway interventions by attenuating both cell death and inflammatory signaling.

These findings may also explain why previous adjunctive therapies targeting individual mediators, such as anti-TNF antibodies, corticosteroids, and pentoxifylline, have failed to improve CM outcomes (*61*–*64*). Endothelial injury appears to be driven by redundant, convergent signals, with multiple death ligands and cytokines activating overlapping receptor pathways. Our single-cell analysis supports this view. While some inflammatory endothelial cells activated apoptotic pathways alone, others simultaneously engaged overlapping regulated cell death programs. RIPK1 integrates inflammatory and death-receptor signals, including TNFR1 and TLR pathways, through its death and RHIM domains (*35*), positioning it as a central node within this network. In vitro, neither RIPK1 nor caspase inhibition alone fully prevented endothelial death, whereas combined inhibition provided near-complete protection. Targeting RIPK1 may therefore intercept shared execution machinery across a heterogeneous and evolving endothelial injury landscape.

Previous studies detected caspase-3 activation in cerebral endothelial cells during both the early and moribund stages of mouse CM (*65*, *66*). Together with our functional data, these observations support a role for regulated endothelial cell death in mouse CM pathogenesis, while its temporal relationship to BBB permeability and its specific contribution to hemorrhagic vascular injury remain unresolved. Infected mice displayed thrombocytopenia and impaired thrombin generation, consistent with disseminated intravascular coagulation-like features in human disease (*12*). While this creates a systemic prohemorrhagic tendency, focal endothelial injury appears to determine where hemorrhages occur. In mice, antigen-specific CD8^+^ T cells arrest on cerebral microvessels and induce localized vascular leakage (*24*, *27*), suggesting that focal endothelial barrier disruption permits blood extravasation when hemostatic capacity is compromised. Region-specific patterns of hemorrhage differed between mice and humans, affecting the brainstem and olfactory bulbs in mice and white matter border zones in humans, but both cascades likely localized to sites of regionally selective vascular injury (*14*–*16*, *42*). Preserving endothelial integrity may therefore limit focal rupture and hemorrhagic amplification, even if permeability also proceeds through death-independent mechanisms (*15*, *67*). Whether RIPK1 inhibition also reduces neurological morbidity requires direct testing.

Human tissue and genetic data provide associative support for pathway relevance beyond the mouse model. Endothelial pRIPK1 was increased in postmortem brain samples from Malawian children with fatal CM, although the tissue analysis did not establish a direct spatial association between CD8^+^ T cells and pRIPK1-positive endothelium. MalariaGEN data showed pathway-level enrichment of regulated cell death loci and genetic association within the RIPK1 network in severe malaria (*48*). Because this analysis was restricted to a predefined RCDdb gene set, it did not evaluate classical human leukocyte antigen (HLA) class I alleles or PRF1, which was not represented in the curated set. These findings support pathway-level convergence across species without establishing that the CD8^+^ T cell–to–RIPK1 mechanism identified in mice operates identically in human CM.

Translating these insights into therapy could be feasible. Several selective RIPK1 kinase inhibitors, including GSK2982772 (*39*) and the CNS-penetrant oditrasertib (SAR443820/DNL788) (*37*), have reached early clinical testing. They generally report favorable short-term tolerability. However, a multiple sclerosis trial with oditrasertib was stopped, as it failed to cause a positive change in NfL compared to placebo (*37*, *68*, *69*)*.* Although clinical efficacy was also limited in a psoriasis study (*39*), this does not preclude a potential benefit in CM, where the pathogenic insult is acute, temporally defined, and driven by a discrete mechanism. A short course of RIPK1 inhibition administered alongside antimalarial therapy could simultaneously suppress parasite replication and prevent immune-mediated vascular injury. Since our in vitro data suggest synergistic protection when RIPK1 inhibition is paired with caspase blockade, combination approaches may also be explored. For use in malaria-endemic settings, candidate RIPK1 inhibitors would also need to be orally deliverable, stable under field conditions, scalable, and affordable.

These findings should be interpreted in the context of several limitations. Our mechanistic dissection relies on the mouse model, which differs from human *P. falciparum* CM in parasite biology, hemorrhage topography, and the balance between sequestration and immune-mediated injury. Even so, PbA-infected erythrocytes accumulate in the brain microvasculature in vivo (*70*) and bind mouse brain microvascular endothelial cells under flow (*71*); the species difference therefore reflects differences in molecular basis and relative pathogenic contribution rather than a complete absence of parasite-endothelial interactions in mice. Cross-species relevance is supported by endothelial pRIPK1 activation in human postmortem tissue and by a polygenic signal in severe malaria GWAS (fig. S5 and table S4). These associative findings support cross-species pathway relevance but do not establish direct mechanistic equivalence. The human data carry additional constraints, as postmortem immunofluorescence captures only fatal cases, the MRI and pathology cohorts derive from different populations and age groups, and the GWAS required a composite severe malaria phenotype because CM-specific case numbers were insufficient for pathway-level detection. The inferred convergent regulated cell death program was based on transcriptomic coactivation rather than biochemical demonstration that these pathways assemble into a dedicated endothelial signaling complex during CM. Endothelial-specific RIPK1 inactivation prolonged survival but did not prevent death, indicating that RIPK1-independent mechanisms contribute to lethality. The RIPK1 inhibitor used in vivo, GNE684, is a tool compound with cross-species activity against human, mouse, and rat RIPK1 (*72*). Translation will require testing with clinically advanced human RIPK1 inhibitors, such as oditrasertib (SAR443820) (*37*), administered within a therapeutically relevant window, rather than from day 1 p.i., as in our experimental design, given that patients with CM invariably present with established disease at the point of coma.

In summary, we identify a RIPK1-regulated endothelial death program linking CD8^+^ T cell cytotoxicity to focal vascular injury in mouse CM. Endothelial genetic inactivation of RIPK1 kinase activity and systemic pharmacological RIPK1 inhibition prolong survival in the mouse model, while human tissue and genetic data provide associative evidence of pathway convergence. These findings support further testing of RIPK1 inhibition as a host-directed adjunctive strategy.

## MATERIALS AND METHODS

### Study design

The primary objective of this study was to identify the molecular mechanisms of brain endothelial cell death during CM and to evaluate RIPK1 as a therapeutic target. The study integrates susceptibility-weighted MRI and LSFM, endothelial-specific translatomics, in vitro cytotoxicity assays, genetic and pharmacological in vivo interventions, human postmortem validation, and genome-wide association analysis. Sample sizes for in vivo experiments were determined on the basis of prior experience with the PbA model and are specified in each figure legend. Mice were randomized to treatment groups; investigators were not blinded to group allocation during experiments, but quantitative analyses were performed using automated pipelines. No data were excluded from analyses. All animal experiments were approved by the relevant institutional authorities and performed in accordance with institutional guidelines. Reagents are listed in table S5.

### Study approval and ethical considerations

All animal experiments received approval from the Hamburg State Authority (Behörde für Justiz und Verbraucherschutz–Amt für Verbraucherschutz, Lebensmittelsicherheit und Veterinärwesen, Freie und Hansestadt Hamburg) and were conducted in accordance with the German Animal Welfare Act (Tierschutzgesetz; TVA N044/2024) and the European Union Directive 2010/63/EU. These experiments were carried out at the Bernhard Nocht Institute for Tropical Medicine (BNITM) and the University Medical Center Hamburg-Eppendorf (UKE), Hamburg, Germany. All procedures adhered to the ARRIVE guidelines for the reporting of animal research. Human MRI studies were performed at Ispat General Hospital, Rourkela, India, and were approved by the institutional ethics committee (IRB no. 21-044). Studies involving human postmortem tissue from pediatric cases of CM in Malawi were approved by the College of Medicine Research Ethics Committee at Kamuzu University of Health Sciences, Blantyre, Malawi, and the University of Liverpool Research Ethics Committee. Written informed consent was obtained from all participants or their legal guardians for every human study.

### MRI of human CM

Human MRI scans were obtained at Ispat General Hospital (IGH), Rourkela, Odisha, India, between October 2013 and May 2024 as part of a study of severe malaria-associated brain changes. Patients with *P. falciparum* malaria were prospectively enrolled with clinical and laboratory assessments. Diagnosis was confirmed by Giemsa-stained blood films and species-specific polymerase chain reaction (PCR); patients with mixed *Plasmodium* infections or concomitant bacterial or viral infections were excluded. Severe malaria was defined according to World Health Organization criteria and further classified as CM on the basis of unarousable coma (Glasgow Coma Scale <11). The analyzed cohort comprised a subset of 10 patients with CM (2 with fatal outcomes) from the Rourkela MRI cohort (*40*) who underwent SWI and 5 healthy controls imaged on the same MRI scanner at IGH (table S1). SWI was used to identify cerebral microhemorrhages, as described in prior Rourkela CM MRI studies.

### Animals

C57BL/6J and OT-I T cell receptor-transgenic mice (C57BL/6-Tg(TcraTcrb)1100Mjb; JAX:003831) were bred in-house under specific pathogen–free (SPF) conditions (12 hours light/dark cycle, 22° ± 1°C, 50 ± 10% humidity). Both sexes, aged 7 to 10 weeks, were used in equal numbers. OT-I mice express a transgenic T cell receptor specific for the OVA-derived peptide OVA257–264 (SIINFEKL) presented by H-2K^b^ and were used as donors of antigen-specific CD8^+^ T cells for the endothelial-cell coculture experiments. *Tie2-L10a-eGFP* transgenic mice were generated by Z.H., University of Wisconsin, USA (*45*). This BAC-TRAP reporter expresses enhanced GFP (eGFP)–tagged L10a and carries no Cre recombinase; it therefore does not mediate gene recombination. *Casp3^fl/fl^* and *Casp7^fl/fl^* were obtained from P.V., VIB, Ghent, Belgium (*73*), and *Mlkl^fl/fl^* was obtained from W. S. Alexander, The Walter and Eliza Hall Institute of Medical Research, Melbourne, Australia (*74*). Slco1c1-CreER^T2^ mice were generated by M.S., University of Lübeck, Germany (*51*). *Slco1c1* is expressed in brain endothelial and choroid plexus epithelial cells rather than hematopoietic lineages, and the driver was reported to recombine selectively in brain endothelium (*51*). The *Ripk1^fl/fl^* (*75*) and *Ripk1^D138N^* (*76*) lines were obtained from M. Pasparakis, University of Cologne, Germany. Endothelial-specific CASP3/7-CKO mice were generated by crossing *Casp3^fl/fl^*;*Casp7^fl/fl^* mice to Slco1c1-CreER^T2^ *mice*; Cre-negative *Casp3^fl/fl^*;*Casp7^fl/fl^* littermates served as controls. Endothelial-specific MLKL-CKO mice were generated by crossing *Mlkl^fl/fl^* mice to *Slco1c1-CreERT2* mice; Cre-negative *Mlkl^fl/fl^* littermates served as controls. RIPK1-KD mice were generated by crossing Slco1c1-CreER^T2^ mice with *Ripk1^fl/fl^*;*Ripk1^D138N^* mice; Cre-negative *Ripk1^D138N/fl^* littermates served as controls. Tamoxifen-induced Cre recombination was achieved via intraperitoneal injection of tamoxifen (75 mg kg^−1^ body weight, dissolved in corn oil) once daily for five consecutive days, 2 weeks before infection. Genotyping was conducted using PCR analysis of ear punch samples. Sample sizes were determined on the basis of pilot studies and existing literature, with a minimum of *n* = 5 to 6 mice per group for survival studies, to detect a 50% difference with 80% statistical power. Each survival curve represents one biologically independent cohort per genotype, in accordance with ARRIVE 2.0 reporting standards and 3Rs principles to minimize animal use in lethal-endpoint experiments.

### PbA infection model

PbA (MRA-311, BEI Resources) parasites were maintained through serial passage in donor C57BL/6J mice and preserved via cryopreservation in Alsever’s solution with 10% glycerol in liquid nitrogen. For experimental purposes, frozen stocks were thawed and subsequently passaged once through naive mice to ensure maximal infectivity. Experimental mice were infected via intraperitoneal injection of 1 × 10^5^ infected RBCs diluted in 200 μl of sterile phosphate-buffered saline (PBS). Control mice received equivalent volumes of uninfected erythrocytes prepared identically. Parasitemia was monitored daily beginning on day 3 p.i. through examination of Giemsa-stained thin blood smears, with approximately 1000 erythrocytes counted per sample. Disease progression was evaluated using the Rapid Murine Coma and Behavior Scale (RMCBS), which assesses gait, balance, motor performance, body position, limb strength, touch escape, pinna reflex, toe pinch, aggression, and grooming, with a maximum score of 20. Mouse CM was characterized by an RMCBS score below 6/20. Mice meeting these criteria or exhibiting severe neurological signs (seizures, ataxia, paralysis, or coma) were euthanized immediately. Monitoring was conducted every 12 hours during the acute phase, specifically days 5 through 10 p.i.

### LSFM and tissue clearing

Mice were perfused with 10 ml of room-temperature PBS, followed by 10 ml of 4% paraformaldehyde (PFA) containing *Lycopersicon esculentum* lectin-DyLight-649 (10 μg ml^−1^) (Vector). Brains were postfixed overnight and optically cleared using the CUBIC tissue-clearing protocol (*77*). Samples were imaged on a mesoSPIM light-sheet microscope (camera Hamamatsu Orca Fusion, detection objective Olympus MVPLAPO-1X, zoom body MVX10). Three-dimensional volumetric images of brain vasculature were acquired from a total of nine mice, comprising uninfected controls (*n* = 4) and PbA-infected animals (*n* = 5), with an effective lateral pixel size of 5.26 μm and an axial z-step of 5 μm. Specimens were optically cleared and imaged in four adjacent tiles (Q1 to Q4), stored in HDF5 format, and reconstructed into contiguous whole-brain volumes by intensity-normalizing each z-slice to 8-bit range and stitching tiles using the OpenCV panoramic stitcher. Anatomical regions of interest, such as the olfactory bulbs, were manually delineated by specifying bounding coordinates along the *x*, *y*, and *z* axes. Voxel-wise segmentation was performed using K-means clustering (scikit-learn), with the optimal number of clusters (*k* = 4) determined via the elbow method by evaluating the sum of squared distances for *k* = 1 to 10. Individual clusters were visually inspected to identify hemorrhage-associated signal, which was isolated as a binary mask. To extend this segmentation across all z-slices, the K-means–derived labels from the reference slice were used as ground truth to train a PyTorch feed-forward neural-network classifier (one intensity input, one 10-unit hidden layer; 80/20 train-test split), which was then applied slice-by-slice to generate a volumetric binary segmentation mask. Total hemorrhage volume was quantified by summing all positively labeled voxels and multiplying by the physical voxel volume (5.26 × 5.26 × 5 = 138.3 μm^3^), enabling direct comparison of hemorrhage burden between control and PbA-infected groups.

### Plasma NfL measurement

Plasma NfL concentrations were quantified in duplicate using a commercially available single-molecule array (Simoa) assay (Neurology 2-Plex B) on the HD-X Analyzer in accordance with the manufacturer’s protocol (Quanterix, Billerica, MA, USA). All assays were conducted in a blinded manner with respect to the experimental groups.

### Electron microscopy and histology

For electron microscopy, mice were euthanized, brains were removed, and brain neocortex was dissected into small pieces (2 mm^3^). The samples were immersion fixed in 4% PFA in 0.1 M phosphate buffer for 48 hours at 4°C. Tissue blocks were washed in 0.1 M phosphate buffer, treated with OsO_4_ [0.5% for 45 min at room temperature (RT)], and stained with uranyl acetate [1% (w/v) in 70% (v/v) ethanol, 45 min at RT]. After dehydration, tissue blocks were embedded in epoxy resin (Durcupan ACM, Sigma-Aldrich, Gillingham, UK). Ultrathin sections (70 nm) were cut using a Leica UC6 ultramicrotome (Leica, Wetzlar, Germany) and analyzed with an 80-kV Zeiss LEO910 transmission electron microscope and Image SP software (TRS-Tröndle, Moorenweis, Germany). For histology, mouse brain tissues were fixed in 4% buffered formalin and processed for paraffin embedding. Sections were cut (2 μm) and stained with hematoxylin and eosin according to standard procedures.

### Hematology in mice

A ProCyte Dx Hematology Analyzer (IDEXX Laboratories) was used to determine complete blood cell counts in citrate-anticoagulated whole blood samples from mice. To test the analyzer’s accuracy, monthly quality control was performed using the manufacturer’s control solution (e-CHECK, XS; IDEXX ProCyte Dx Quality Control). Complete blood cell counts were measured within 2 hours of sampling.

### Real-time thrombin generation in mouse plasma

Mice were deeply anesthetized by intraperitoneal injection of an aqueous solution containing esketamine hydrochloride (10 mg ml^-1^; Pfizer) and xylazine hydrochloride (1.6 mg ml^−1^; Bayer), at 100 μl per 10 g body weight, corresponding to 100 and 16 mg kg^−1^, respectively. Mouse whole blood was collected by cardiac puncture into tubes containing 0.106 M trisodium citrate. The anticoagulated whole blood was centrifuged at 150*g* for 10 min to obtain platelet-rich plasma (PRP). Preparation of platelet-poor plasma (PPP) from PRP involved two consecutive centrifugation steps at 1500*g* for 10 min, with the second centrifugation serving to remove residual cells and cellular debris. All steps of plasma preparation were performed at RT. Thrombin generation in mouse PPP was analyzed by the calibrated automated thrombogram technique, based on a modified protocol described by Tchaikovski *et al.* (*78*). Assays were conducted in transparent 96-well round-bottom plates (no. 650101, Greiner Bio-One) with a final reaction volume of 60 μl. To initiate coagulation activation, 20 μl of PPP was added to one of the following mixtures: (i) PPP-Reagent LOW, containing tissue factor (1 pM) and phospholipids (4 μM), (ii) kaolin (10 μg ml^−1^) supplemented with the phospholipid containing MP-Reagent, or (iii) MP-Reagent as a phospholipid-only control to determine unstimulated baseline activity in plasma. Tris buffer (20 mM, pH 7.4) was used to achieve an intermediate volume of 50 μl in all wells. Reaction mixtures were then equilibrated for 10 min at 33°C using a Fluoroskan Ascent microplate reader (Thermo Fisher Scientific, Waltham, USA). Thrombin generation was initiated after the reader automatically dispensed 10 μl of the FluCa solution to each well. The Thrombinoscope software package (version 5.0.0.742) automatically processed fluorescence signals detected at an excitation of 390 nm and at an emission of 460 nm into a thrombogram representing thrombin concentrations over time. These calculations include corrections for inner filter effects and substrate artifacts by referencing an α2-macroglobulin-thrombin calibrator of known activity, which was added to the plasma samples instead of a coagulation-activating reagent. All experimental conditions and the corresponding calibrator reference were measured in duplicate.

### Translating ribosome affinity purification sequencing

Endothelial cell–specific translatome profiling was performed using *Tie2-L10a-eGFP* transgenic mice (*45*), which express an EGFP-tagged ribosomal protein L10a under control of the endothelium-specific *Tie2* (Tek) promoter (*45*). Mice were infected with PbA as described above (mock, *n* = 5; PbA, *n* = 6; PbA + αCD8, *n* = 6). On day 6 p.i., animals were transcardially perfused with ice-cold PBS containing cycloheximide (100 μg ml^−1^) to arrest translation and stabilize ribosome-mRNA complexes. Brains were rapidly dissected and homogenized in ice-cold polysome lysis buffer [20 mM Hepes (pH 7.4), 150 mM KCl, 10 mM MgCl_2_, 0.5 mM dithiothreitol, cycloheximide (100 μg ml^−1^), EDTA-free protease inhibitors, RNase inhibitor] using a Dounce homogenizer. Lysates were cleared by centrifugation (2000*g*, 10 min, 4°C), then NP-40 (1%) and DHPC (30 mM) were added. A second clarification (20,000*g* for 10 min at 4°C) yielded the postmitochondrial supernatant. GFP-tagged polysomes were immunoprecipitated using anti-GFP nanobody-coupled paramagnetic beads (GFP-Trap, Chromotek) with rotation for 30 min at 4°C. Beads were washed four times with high-salt wash buffer (350 mM KCl) to reduce background. RNA was eluted and purified using RNeasy Micro kits (Qiagen). Sequencing libraries were prepared using the TruSeq RNA Library Prep Kit v2 (Illumina) and sequenced on an Illumina HiSeq 4000 [1 × 50 base pairs (bp)]. Reads were aligned to GRCm38 using STAR 2.5.2b, and gene-level counts were generated with featureCounts 1.5.1. Differential expression analysis was performed with DESeq2 1.40 (Benjamini-Hochberg FDR < 0.05). Gene set enrichment analysis was performed using fgsea (1000 permutations) against the Hallmark, Reactome, and Gene Ontology (GO) databases.

### Brain-infiltrating CD8^+^ T cell isolation and RNA-seq

Mice were perfused transcardially with ice-cold PBS containing 0.05% EDTA. Brains were minced and digested with collagenase D (1 mg ml^−1^) and DNase I (0.5 mg ml^−1^) for 30 min at 37°C with gentle agitation. Single-cell suspensions were passed through a 70-μm strainer, resuspended in 30% isotonic percoll, and centrifuged at 500g for 10 min at 4°C (brake off). Pellets were subjected to RBC lysis (tris-ammonium chloride buffer) and stained with anti-CD3ε-APC, anti-CD8α-FITC, and anti-CD44-PE-Cy7 (BioLegend). CD3^+^CD8^+^CD44^+^ cells were sorted on a BD FACSAria Fusion to >98% purity. For splenic comparator samples, spleens from mock-infected and infected mice were mechanically dissociated through 100-μm nylon mesh, subjected to erythrocyte lysis, filtered, and stained with the same antibody panel before sorting CD3^+^CD8^+^CD44^+^ cells under identical conditions. RNA was eluted and purified using RNeasy Micro kits (Qiagen). Sequencing libraries were prepared using the Stranded Total RNA Prep Kit (Illumina) and sequenced on an Illumina NovaSeq 6000 (2 × 50 bp). Reads were mapped to GRCm38 with STAR 2.7.11; gene counts were generated with featureCounts 1.5.1. The unpaired brain-versus-infected-spleen contrast was tested using DESeq2 1.40 with Benjamini-Hochberg correction (FDR < 0.05); row-scaled heat maps were used only for visualization and not for gene-level inference. Hallmark, Reactome, and GO pathway enrichment were performed using fgsea (1000 permutations). Ligand-receptor interaction inference was performed using the BulkSignalR R package. Bulk RNA-seq count matrices from brain endothelial TRAP-seq and brain-infiltrating CD8^+^ T cells were merged into a single expression matrix. Mouse gene symbols were converted to human orthologs using the findOrthoGenes function to enable querying of the BulkSignalR ligand-receptor database. Statistical parameters were learned from reference-tissue expression data (learnParameters), and ligand-receptor pairs were inferred on the basis of Spearman correlation with significance assessed against empirical null distributions (Benjamini-Hochberg–adjusted *q* < 0.001). Pathway-level enrichment of inferred interactions was evaluated against Reactome gene sets, with results reduced to best-pathway assignments (reduceToBestPathway) and visualized as bubble plots (bubblePlotPathwaysLR). Published mouse CM single-cell RNA-seq data (*46*) were reanalyzed by inflammatory module scoring and endothelial reclustering. Endo_Inflammatory was defined as an analysis-derived state marked by *Vcam1*, *Ccl3*, and *Ccl4*; abundance was calculated per biological replicate as a fraction of endothelial cells, and group differences were tested by exact permutation Kruskal-Wallis.

### Mouse brain immunohistochemistry and quantification

For mouse cell-death marker quantification, control and day 6 PbA-infected mice were transcardially perfused with PBS followed by 4% PFA. Brains were postfixed for 4 hours, stored in PBS, dehydrated, paraffin embedded, and sectioned at 2-μm thickness. Sections were deparaffinized and stained using a Ventana BenchMark XT autostainer (Ventana, Tucson, AZ, USA). After dewaxing and inactivation of endogenous peroxidases (3% hydrogen peroxide), sections were blocked and incubated overnight at 4°C with primary antibodies against cleaved caspase 3 (MAB835, R&D Systems) or phosphorylated MLKL (ab196436, Abcam). Specific binding was visualized using DAB with the Histofine Simple Stain MAX PO Universal immunoperoxidase polymer kit (Nichirei Biosciences, Wedel, Germany). Counterstaining and bluing were performed with hematoxylin (Ventana Roche, no. 760-2021) and Bluing Reagent (Ventana Roche, no. 760-2037) for 4 min. Stained sections were mounted in mounting medium. The cortex, brainstem, hippocampus, and olfactory bulb were evaluated in anatomically matched sagittal sections (fig. S4). Images were acquired under identical settings using a NanoZoomer S20MD slide scanner system (C16300-21MDEU, Hamamatsu, Japan). Using QuPath (Open source software, v.0.5.1), DAB-positive cells were manually identified in the cortex, brainstem, hippocampus, and olfactory bulb. Two anatomically matched sections per animal were averaged to generate one biological replicate, normalized to the respective region area, and reported as positive cells per square millimeter. Quantification was performed by an investigator blinded to the experimental group.

### Human postmortem tissue immunofluorescence

Formalin-fixed paraffin-embedded brain tissue sections from Malawian patients with fatal CM (*n* = 8) and encephalopathic illness (*n* = 8) were stained using a multiplex Tyramide Signal Amplification system (table S2). Cortical sections (5 μm thick) were dewaxed and rehydrated through xylene and a graded alcohol series. Heat-induced antigen retrieval was performed as follows: CD31, in citrate buffer (pH 6.0) with microwave heating; fibrinogen, in EDTA buffer (pH 9.0) with microwave heating; and phospho-RIPK1, in citrate buffer (pH 6.0) with pressure-cooker heating. Sections were then incubated with BLOXALL Endogenous Blocking Solution (SP-6000-100, Vector Laboratories), followed by FcR Blocking Reagent (130-059-901, Miltenyi Biotec), and a blocking solution (10% horse serum, 5% human serum, 5% bovine serum albumin, in 1× TBST). Overnight incubation (4°C) was performed with the following primary antibodies: rabbit anti-CD31 (NB100-2284, Novus Biologicals; 0.05 μg ml^−1^), goat anti-fibrinogen (PA1-26809, Invitrogen; 4.5 μg ml^−1^), and rabbit anti–phospho-RIPK1 (44590S, Cell Signaling Technology; 0.38 μg ml^−1^). Bound primary antibodies were detected using ImmPRESS-HRP horse anti-rabbit IgG Polymer Detection Kit (MP-7401, Vector Laboratories) or ImmPRESS-HRP horse anti-goat IgG Polymer Detection Kit (MP-7405, Vector Laboratories), as appropriate, and visualized with reactive Opal fluorophores (NEL811001KT, Akoya Biosciences). Isotype controls were included to confirm specificity. Slides were digitized at ×40 magnification using a NanoZoomer S60 digital slide scanner (C13210-01, Hamamatsu Photonics) and NZConnect 1.1.0 software (Hamamatsu Photonics).

### Human genome analysis

Genome-wide association data from the MalariaGEN consortium were analyzed across three African cohorts (Gambia, *n* = 5593; Kenya, *n* = 3652; Malawi, *n* = 3088), comprising 6341 severe malaria cases and 5992 controls (*79*). Samples were excluded for high missingness, sex discrepancies, or outlying heterozygosity; variants were filtered for missingness, deviation from Hardy-Weinberg equilibrium, and minor allele frequency below 0.01. Case-control association testing was performed within each cohort using logistic regression under an additive genetic model (PLINK 2.0; www.cog-genomics.org/plink/2.0/) (*80*), with the first 10 principal components as covariates to account for population stratification. A curated regulated cell death gene set (622 genes, 214,514 variants) encompassing regulators of regulated cell death was compiled from the RCDdb (*47*). The analysis was restricted to variants mapping to this predefined gene set; *PRF1* was not included, and classical *HLA* alleles were not imputed. Pathway-level enrichment was evaluated by comparing the genomic inflation factor (λGC) of regulated cell death variants to the genome-wide background, and its significance was assessed in the Gambian discovery cohort by single nucleotide polymorphism–label permutation testing (10,000 permutations). For the *RIPK1* locus, linkage disequilibrium was computed from the 1000 Genomes Project African (AFR) reference panel (GRCh38) (*81*, *82*). Mendelian randomization was performed using the inverse-variance weighted method (*83*) [TwoSampleMR R package; (*84*)] with whole-blood eQTL data from the eQTLGen consortium as exposure (*85*); genome-wide significant variants (*P* < 5 × 10^−8^) served as instruments. Sensitivity analyses included MR-Egger regression (*86*) to assess directional pleiotropy and Cochran’s Q test to assess instrument heterogeneity.

### In vitro CTL-mediated endothelial killing assay

Primary brain microvascular endothelial cells (PMBECs) were isolated from C57BL/6J mice as described (*87*) with modifications. Briefly, brains were homogenized, separated by dextran density centrifugation (6080*g* for 10 min at 4°C), and then enzymatically digested for 90 min at 37°C. Microvascular fragments were seeded onto rat tail collagen I–coated (50 μg ml^−1^ in 0.02 N acetic acid) opaque-walled flat-bottom 96-well plates and cultured using puromycin selection to enrich endothelial cells (*23*). Cells were used ≥7 days postseeding. Forty-eight hours before coculture, PMBECs were stimulated with recombinant mouse interferon-γ (10 ng ml^−1^) for 24 hours to induce MHC class I expression; medium was then replaced with fresh medium containing chicken OVA (0.3 mg ml^−1^), and cells were incubated for an additional 24 hours to allow processing and presentation of the OVA_257–264_ (SIINFEKL) epitope onto H-2K^b^ (*88*, *89*). Effector cells were obtained from OT-I mice. OT-I donor mice were infected with 1 × 10^5^ erythrocytes infected with an OVA-expressing PbA line (PbTG) 6 days before the coculture assay (*50*). On day 6 after infection, spleens were collected and mechanically dissociated through 100-μm nylon mesh, subjected to erythrocyte lysis (5 min, RT), and sequentially filtered through 70- and 30-μm strainers. Splenocytes were incubated with Fc block and stained with anti-CD3, anti-CD8, and anti-CD44 (each at 1:100 dilution) for 20 min at 4°C in the dark. Activated CD3^+^CD8^+^CD44^+^ CTLs were sorted on a BD FACSAria (85-μm nozzle, 4°C) to ≥95% purity and resuspended in RPMI 1640 complete medium supplemented with IL-2 (50 U ml^−1^). For coculture, pharmacological compounds were diluted in RPMI complete medium, added to PMBEC monolayers, and preincubated for 2 hours at 37°C, 5% CO_2_. After preincubation, medium was removed, CTLs were added at an effector-to-target ratio of 1:9, and compounds were re-added to a final volume of 200 μl per well. Plates were incubated for 20 hours at 37°C, 5% CO_2_. Following coincubation, wells were washed gently three times with warm PBS, and viability was quantified using the CellTiter-Glo Luminescent Cell Viability Assay (Promega): 100 μl of RPMI complete medium and 100 μl of CellTiter-Glo reagent were added per well, plates were shaken orbitally for 120 s, equilibrated for 10 min at RT, and luminescence was measured on a TECAN plate reader.

### Assessment of Slco1c1-CreER^T2^ recombination specificity

To assess CreER^T2^-mediated recombination in brain and hematopoietic compartments, Slco1c1-CreER^T2^ mice were crossed with *Gt(ROSA)26Sor*^*tm1Sor*^ *(Gt(ROSA)26Sor)* reporter mice, as described previously (*51*, *90*). Compound reporter mice received tamoxifen (Sigma-Aldrich; 1 mg per dose) every 12 hours for five consecutive days. β-Galactosidase activity was assessed histochemically in 40-μm cryosections of brain and spleen and peripheral blood and bone marrow smears. Samples were counterstained with eosin as described previously (*91*).

### Pharmacological treatments in mice

For RIPK1 inhibition, GNE684 (Genentech) (*72*) was dissolved at 10 mg ml^−1^ in vehicle consisting of 10% dimethyl sulfoxide, 40% polyethylene glycol, molecular weight 400, 5% Tween 80, and 45% double-distilled water. Mice received GNE684 or vehicle (20 mg kg^−1^) by oral gavage twice daily (every 12 hours), beginning on day 1 p.i. and continuing through day 10 p.i. or until endpoint. Twice-daily oral administration of GNE684 across doses of 1 to 50 mg kg^−1^ has previously shown in vivo activity in mouse inflammatory disease models (*72*); here, mice received 20 mg kg^–1^ per dose. For CD8^+^ T cell depletion, anti-mouse CD8α monoclonal antibody (clone 2.43, BioXcell, no. BP0061) was administered at 250 μg per mouse by intraperitoneal injection on days 1 and 3 p.i. Control mice received equivalent doses of rat IgG2b isotype control antibody (BioXcell). Depletion efficiency (>95%) was confirmed by flow cytometry of peripheral blood samples on day 6 p.i.

### Statistical analysis

Data are shown as means ± SEM. Two-group comparisons used a two-tailed unpaired *t* test or Mann-Whitney *U* test, as appropriate on the basis of Shapiro-Wilk normality testing. Within-mouse comparisons of regional hemorrhagic burden in Fig. 2J used a two-tailed paired *t* test. Multiple group comparisons used one-way or two-way analysis of variance (ANOVA) followed by Holm-Šidák’s post hoc test. Survival curves were compared using log-rank (Mantel-Cox) tests. Correlations between hematological parameters and disease severity scores (Fig. 3B) were assessed using two-sided Spearman rank correlation. Sample-to-sample transcriptomic correlations (Fig. 5C) and BulkSignalR analyses (Fig. 5, E to G) also used Spearman rank correlation, whereas the normalized enrichment score comparison in Fig. 4E used Pearson’s *r*. Group separation based on PC1 and PC2 scores among endothelial-enriched IP samples was assessed using exact-permutation PERMANOVA, followed by pairwise tests with Holm adjustment; homogeneity of multivariate dispersion was assessed separately. The anti-CD8 centroid shift was expressed as a fraction of the PbA-to-mock centroid distance with a 95% confidence interval. Endo_Inflammatory abundance was compared using an exact-permutation Kruskal-Wallis test. Analyses were performed in GraphPad Prism 11 and R; *P* < 0.05 was considered statistically significant. For survival studies, each Kaplan-Meier curve represents one biologically independent cohort per genotype, prospectively sized for the primary endpoint.
