# Supplementary material for: CD8+ T cells induce RIPK1-regulated brain endothelial cell death in cerebral malaria
Source: Sci Adv. 2026 Nov 6;12(45):eaej4381. doi: 10.1126/sciadv.aej4381 (PMC13742144; doi:10.1126/sciadv.aej4381)
Supplement: Supplementary file 1 [file sciadv.aej4381_sm.pdf]

Science Advances
AAAS

Supplementary Materials for

CD8$^+$ T cells induce RIPK1-regulated brain endothelial cell death in cerebral malaria

Alexandros Hadjilaou *et al.*

Corresponding author: Thomas Jacobs, tjacobs@bnitm.de; Manuel A. Friese, manuel.friese@zmnh.uni-hamburg.de

Sci. Adv. **12**, eaej4381 (2026)
DOI: 10.1126/sciadv.aej4381

The PDF file includes:

Figs. S1 to S6
Tables S1 to S5
Legends for movies S1 to S4
Legend for data S1

Other Supplementary Material for this manuscript includes the following:

Movies S1 to S4
Data S1

SUPPLEMENTARY FIGURES

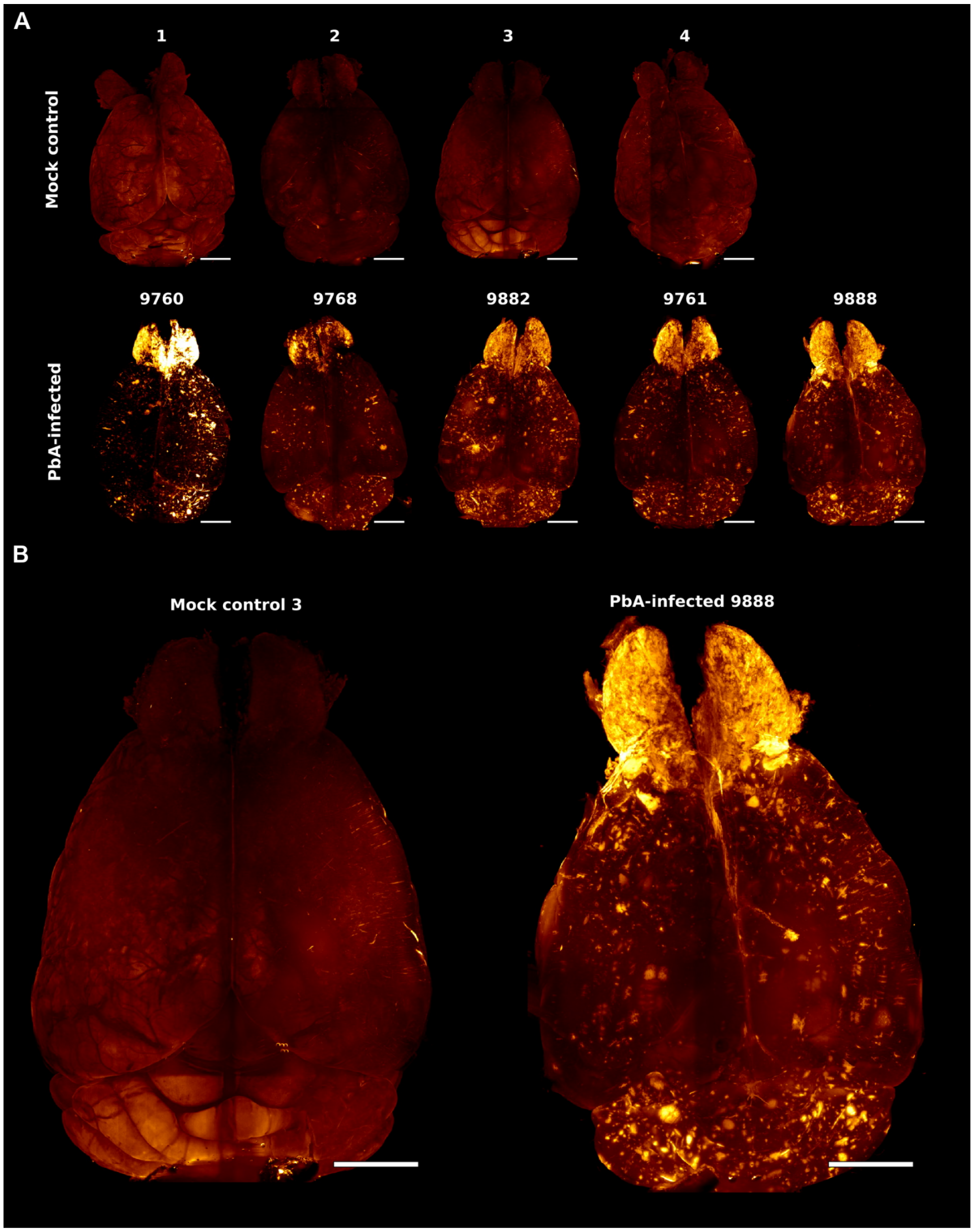

Fig. S1. Whole-brain LSFM comparison of control and PbA-infected mice. (**A**) Dorsal maximum-intensity projections of the tomato lectin 647-nm channel from mock-infected control brains (n = 4) and PbA-infected brains (n = 5) used for volumetric analysis. (**B**) Higher-resolution maximum-intensity projections from representative mock-infected control 3 (left) and PbA-infected mouse 9888 (right). The same linear intensity window (300–2800 arbitrary units; $\gamma = 1.0$) and pseudocolor lookup table were applied to all images. Olfactory bulbs are oriented upward. Scale bars, 2 mm.

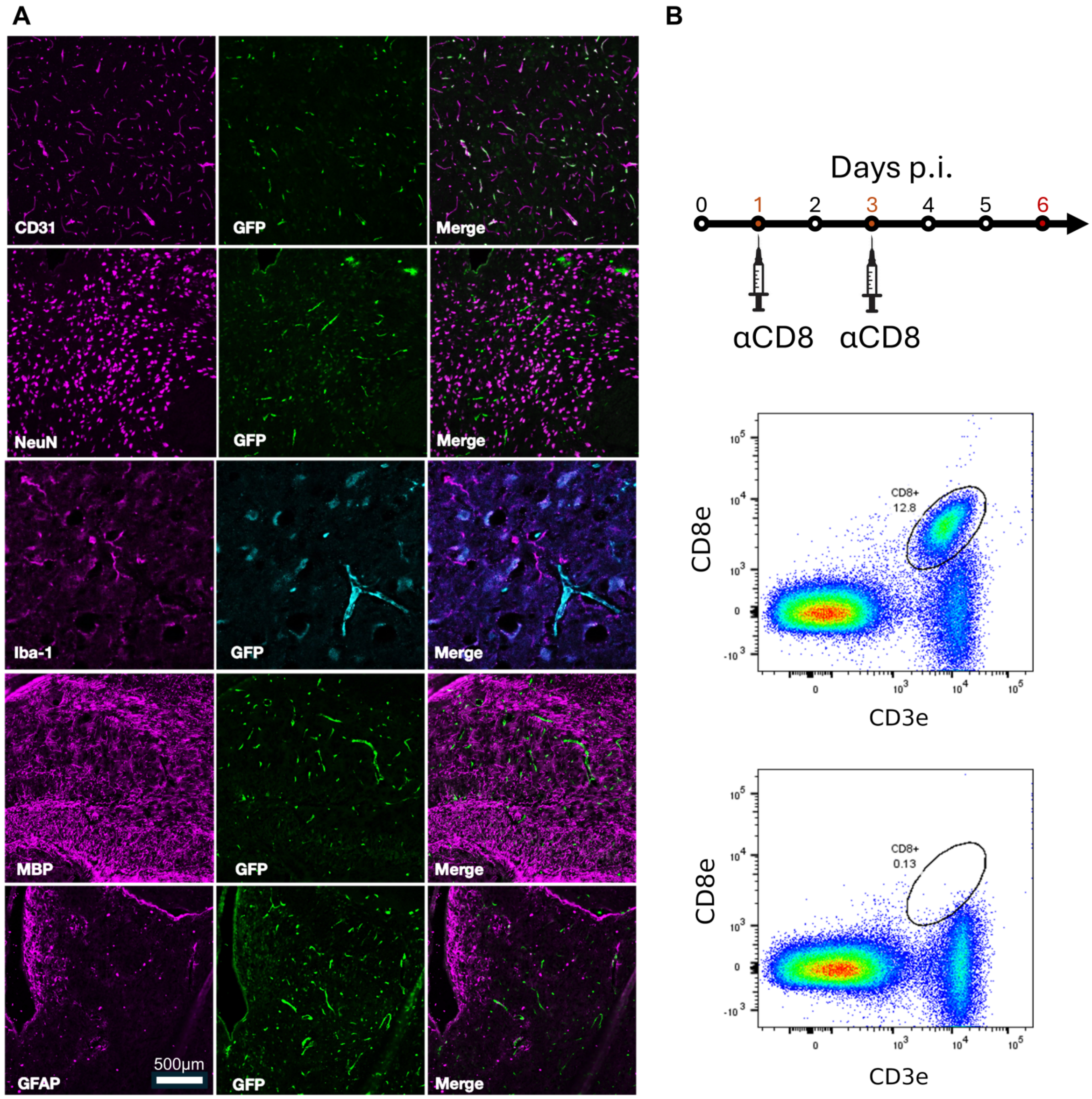

Fig. S2. Validation of endothelial ribosome tagging and CD8⁺ T cell depletion. (**A**) Immunofluorescence validation of GFP expression in *Tie2-L10a-eGFP* transgenic mice. GFP (green) colocalized exclusively with CD31⁺ endothelial cells (top row) but not with NeuN⁺ neurons (second row), Iba-1+ microglia (third row), MBP⁺ oligodendrocytes (fourth row), or GFAP⁺ astrocytes (bottom row). Merged images include DAPI nuclear stain (blue). Scale bar: 500 μm. Representative of n = 3 mice. (**B**) Flow cytometric validation of CD8⁺ T cell depletion protocol. Top: Timeline showing anti-CD8 antibody (αCD8) administration on days 1 and 3 post-infection (p.i.). Middle and bottom: Representative flow cytometry plots showing $CD3^{+}CD8^{+}$ populations in peripheral blood at day six. CD8⁺ T cells comprised 12.8% of lymphocytes in untreated infected mice, but were reduced to 0.13% following depletion, confirming >98% depletion efficiency. Gates show $CD8^{+}CD4^{-}$ populations.

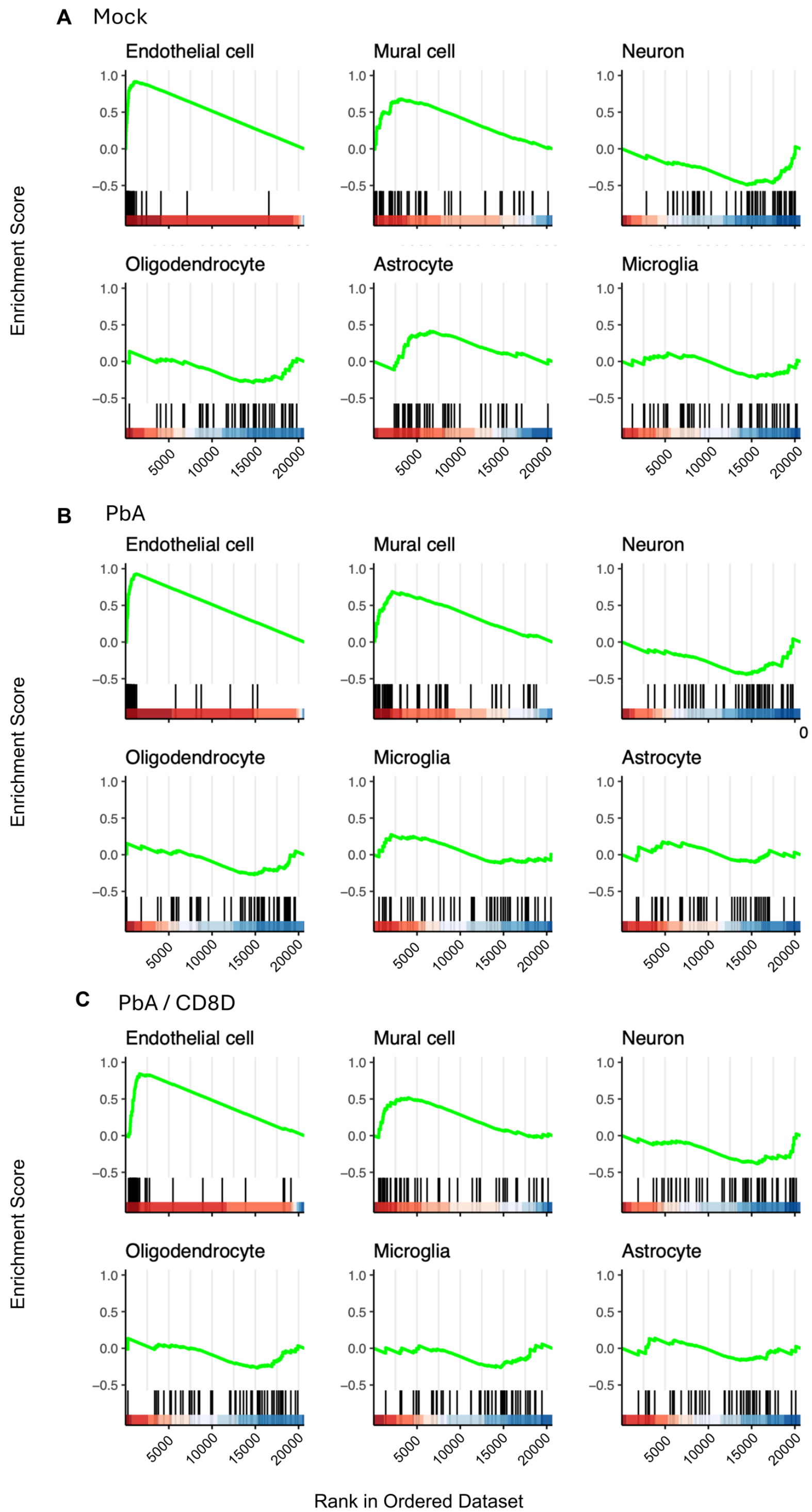

Fig. S3. Gene set enrichment analysis of endothelial cell-specific TRAP-seq. (**A**) Cell type-specific marker enrichment in mock-infected mice. Gene set enrichment analysis (GSEA) plots show strong positive enrichment of endothelial cell markers (enrichment score, ES = 0.8) and depletion of markers for mural cells, neurons, oligodendrocytes, microglia, and astrocytes (ES = −0.5 to −0.8) in immunoprecipitated (IP) versus input control (IC) samples. (**B**) Similar enrichment patterns were observed in PbA-infected mice, confirming endothelial specificity despite inflammation. (**C**) CD8$^+$ T cell depletion (PbA + CD8D) preserved endothelial enrichment while preventing inflammatory changes. *n* = 5–6 mice per group. Ranked gene lists are shown below each plot, with leading-edge genes indicated in black bars.

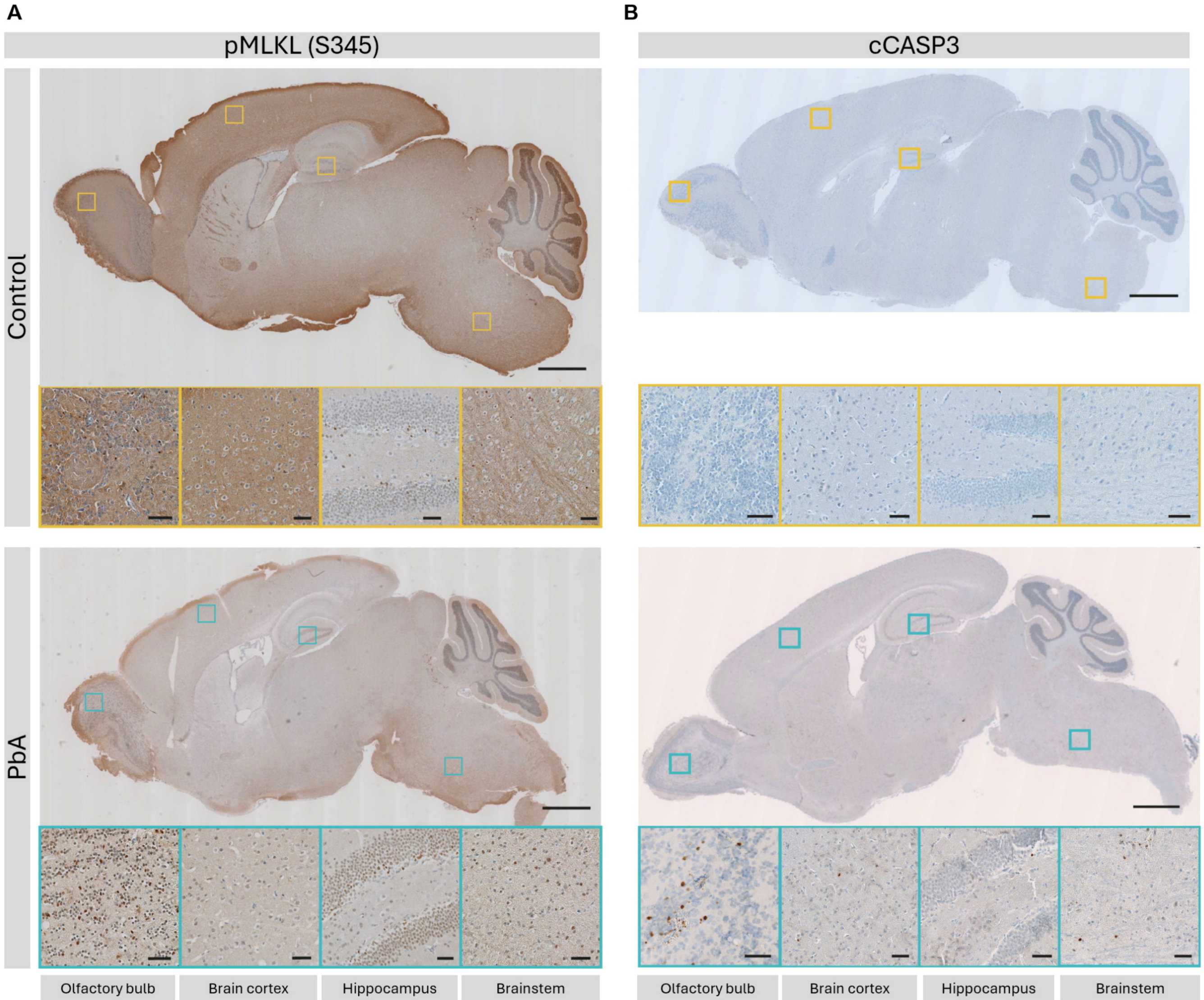

Fig. S4. Representative immunohistochemistry for cell death markers in mouse cerebral malaria. (**A**, **B**) Representative sagittal brain sections from control and PbA-infected mice stained by DAB-based chromogenic immunohistochemistry for (A) phosphorylated MLKL (pMLKL, S345) and (B) cleaved caspase 3 (cCASP3). Boxed regions indicate the olfactory bulb, brain cortex, hippocampus, and brainstem used for high-magnification imaging and quantification in Fig. 6. Scale bars: 1 mm in overview images; 50 µm in high-magnification images.

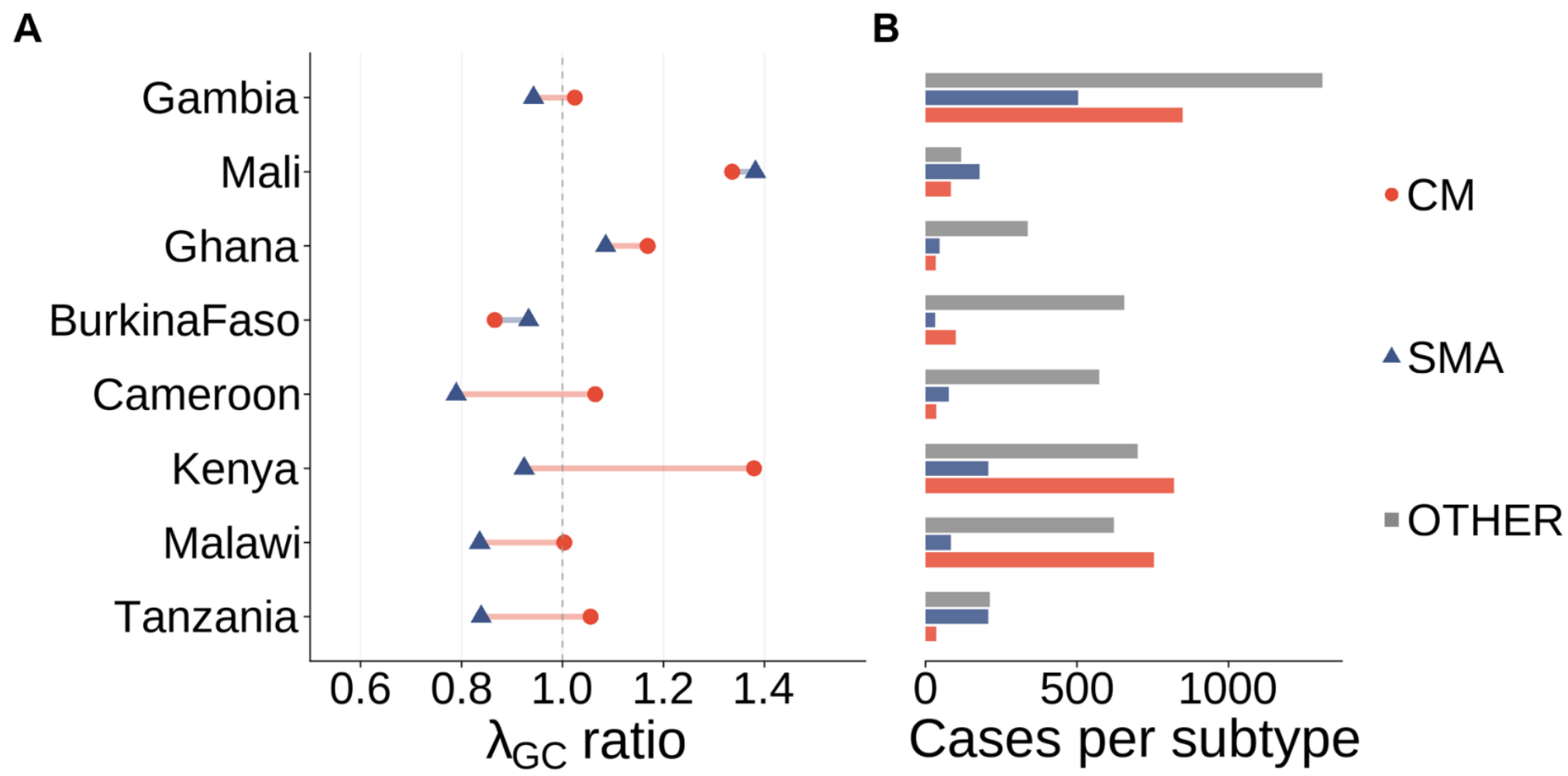

Fig. S5. Subphenotype-stratified regulated cell death pathway enrichment across eight MalariaGEN cohorts. (**A**) Regulated cell death pathway enrichment ratios (λ_GC pathway / λ_GC background) from independent Firth logistic regression GWAS using cerebral malaria (CM)-only (red circles), severe malaria anemia (SMA)-only (blue triangles), or other severe malaria cases (grey squares) versus population controls. Connecting segments link CM and SMA within each cohort. Dashed line indicates no enrichment (ratio = 1). CM showed regulated cell death pathway enrichment above the genome-wide background in 6 of 8 cohorts. Three discovery cohorts (Gambia, Kenya, Malawi) correspond to those analyzed in Fig. 7; five validation cohorts (Mali, Ghana, Burkina Faso, Cameroon, Tanzania) represent independent MalariaGEN populations. (**B**) Number of cases per severe malaria subtype and cohort, illustrating the 2–3-fold reduction in effective sample size that limits statistical power for subphenotype-stratified pathway analyses relative to the composite severe malaria phenotype used in Fig. 7 (6,341 cases).

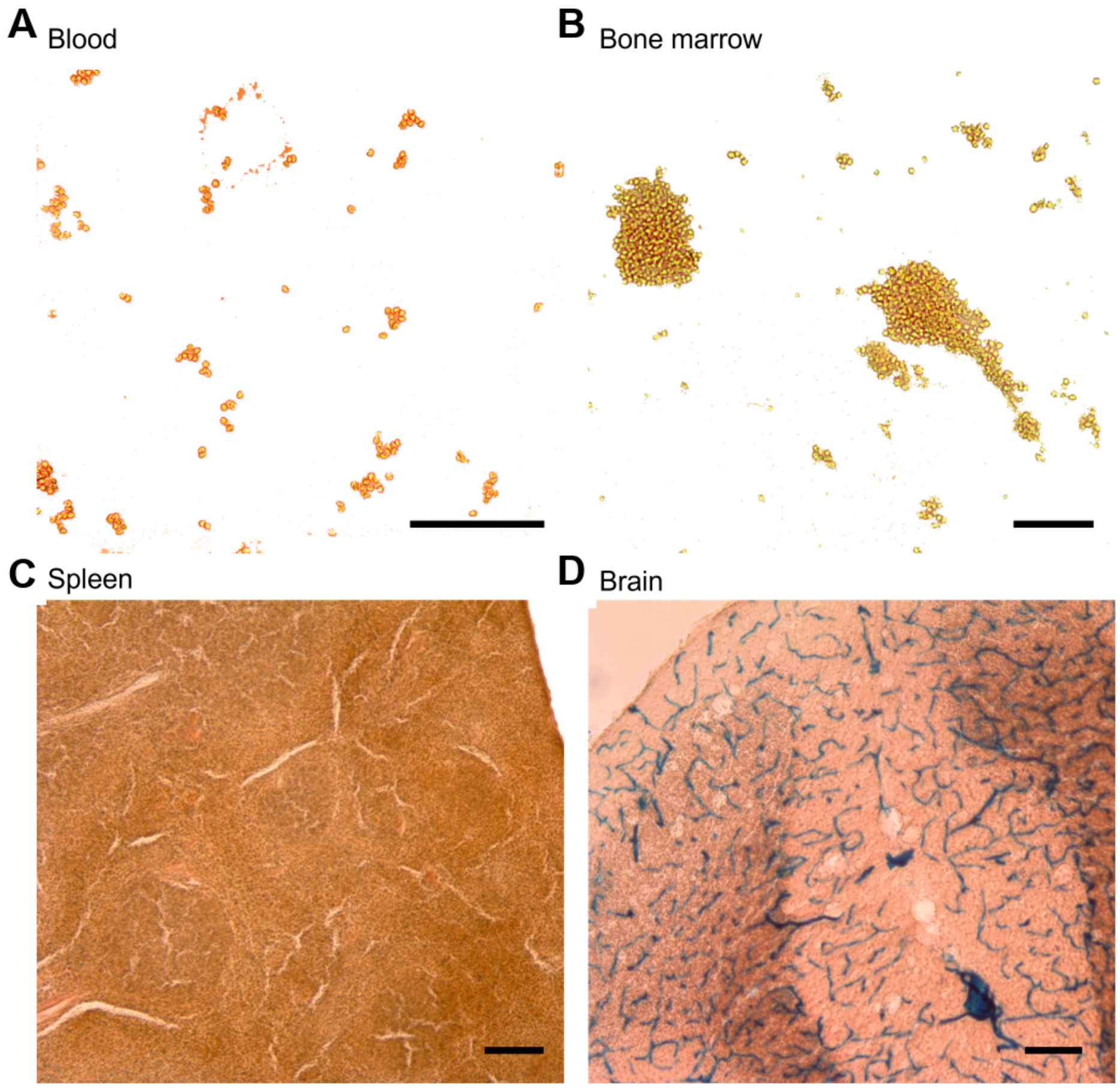

Fig. S6. Slco1c1-CreERT2 reporter activity in brain and hematopoietic compartments. (**A**–**D**) Histochemical β-galactosidase staining of peripheral blood smears (A), bone marrow smears (B), spleen cryosections (C), and brain cryosections (D) from tamoxifen-treated *Slco1c1*-Cre^{ERT2};*Gt(ROSA)26Sor* reporter mice. Reporter LacZ activity was detected in cerebral vessels but not in peripheral blood, bone marrow, or spleen. Samples were counterstained with eosin. Scale bars: 100 *µ*m.

SUPPLEMENTARY TABLES

Table S1. Clinical characteristics of cerebral malaria patients presenting with cerebral microhemorrhages. The cerebral malaria patients were drawn from the Rourkela MRI cohort previously described by Jin et al. *(40)*.

**Cerebral malaria group**					
**Subject**	**Age**	**Sex**	**Parasitemia (parasites per µL)**	**PfHRP2 (ng mL^{-1})**	**GCS**
1	24	M	240	148.7	7
2	32	M	11,390	ND	5
3	22	M	11,729	11.2	5
4	5	M	13,769	14.94	4
5	5	M	79	7.49	5
6	47	M	1,008	10.34	3
7	25	F	391	ND	8
8	9	M	400	1827	6
9	5	M	2,728	1048.4	8
10	26	M	169	686.8	8
**Control group**					
1	34	F			
2	34	M			
3	42	M			
4	24	F			
5	33	M			

PfHRP2 = Plasmodium falciparum histidine-rich protein 2 antigen; GCS = Glasgow Coma Scale

Table S2. Clinical characteristics of pediatric cerebral malaria fatalities and non-malaria controls included in postmortem immunofluorescence analysis.

Patient	Group	Clinical diagnosis before postmortem	Age (months)	Sex	Person living with HIV	Parasitemia before death (parasites per µL)
1	Non-CM control	Meningitis	7	F	No	0
2	Clinical CM	CM2	30	M	No	45,358
3	Clinical CM	CM2	20	M	No	1,174,980
4	Clinical CM	CM2	61	F	No	424,000
5	Non-CM control	Meningitis and Reye's syndrome	29	F	Not tested	0
6	Clinical CM	CM2 and SMA	37	F	Yes	275,200
7	Non-CM control	Salicylate toxicity	33	F	No	114
8	Non-CM control	Pneumonia	144	F	Yes	0
9	Non-CM control	SMA and Reye's syndrome	49	F	No	164
10	Non-CM control	Reye's syndrome	121	M	Yes	0
11	Non-CM control	Reye's syndrome	19	M	No	0
12	Clinical CM	CM2	14	F	No	820
13	Non-CM control	Sepsis (Salmonella)	96	F	No	0
14	Clinical CM	CM2	144	M	Yes	36,250
15	Non-CM control	Sepsis (Salmonella) and TB	36	F	Yes	0
16	Non-CM control	Small bowel obstruction	7	M	No	66,844
17	Clinical CM	CM2	15	M	No	637,000
18	Clinical CM	CM2	31	M	No	0
19	Clinical CM	CM2	26	F	No	6,000
20	Clinical CM	CM2	42	F	No	364

CM, cerebral malaria; CM2, cerebral malaria pathological phenotype 2; SMA, severe malaria anemia; TB, tuberculosis; F, female; M, male; HIV, human immunodeficiency virus. CM2 represents the Dorovini-Zis pathological subtype of cerebral malaria, in which infected red blood cell sequestration is accompanied by intravascular microthrombi and perivascular ring hemorrhages *(9)*.

Table S3. Differential Expression of Genes Displayed in Figure 5d Between Brain-Infiltrating and Splenic CD44^{hi}CD8^{+} T Cells During *Plasmodium berghei* ANKA Infection

Gene	Brain PbA, *n* = 3; mean (SD)	Spleen PbA, *n* = 5; mean (SD)	Spleen mock, *n* = 5; mean (SD)	log2 fold change (95% CI)	Wald z	*P* value	FDR *q* value	Inference at FDR 0.05
Nkg7	5961.6 (620.7)	4950.0 (561.5)	1988.5 (213.4)	0.27 (0.06, 0.48)	2.54	.011	.048	Higher in brain
Prf1	4375.2 (152.6)	2943.0 (419.1)	670.3 (56.2)	0.57 (0.37, 0.77)	5.60	2.17e-8	7.82e-7	Higher in brain
Gzmb	42195.2 (2947.8)	27727.6 (3582.2)	193.3 (232.6)	0.61 (-0.76, 1.97)	0.87	.386	.561	No evidence of differential expression
Gzmk	2257.7 (355.2)	2391.9 (266.7)	73.2 (75.0)	-0.08 (-1.41, 1.24)	-0.12	.902	.944	No evidence of differential expression
Ifng	3022.2 (1444.4)	567.7 (43.9)	133.7 (48.1)	2.41 (1.77, 3.05)	7.37	1.66e-13	1.77e-11	Higher in brain
Stat1	1063.8 (146.9)	1458.0 (181.1)	1211.7 (112.0)	-0.45 (-0.68, -0.23)	-3.94	8.15e-5	9.61e-4	Lower in brain
Irf1	2026.3 (201.4)	2342.6 (95.8)	1934.9 (83.3)	-0.21 (-0.34, -0.08)	-3.09	.002	.013	Lower in brain
Cxcl10	27.7 (9.6)	24.6 (4.7)	8.0 (3.8)	0.18 (-0.53, 0.89)	0.49	.622	.759	No evidence of differential expression
Gbp2	793.5 (43.3)	1197.8 (183.4)	351.4 (35.5)	-0.59 (-0.83, -0.36)	-4.96	6.93e-7	1.61e-5	Lower in brain
Pdcd1	365.7 (87.1)	252.5 (20.2)	37.5 (25.6)	0.53 (-0.02, 1.09)	1.89	.058	.158	No evidence of differential expression
Lag3	1204.9 (88.9)	1267.7 (105.3)	79.2 (51.3)	-0.07 (-0.84, 0.69)	-0.19	.851	.913	No evidence of differential expression
Havcr2	1682.2 (88.3)	1036.5 (99.7)	41.0 (43.2)	0.70 (-0.74, 2.13)	0.95	.340	.519	No evidence of differential expression
Tox	44.9 (8.6)	89.3 (13.9)	584.8 (103.7)	-1.00 (-1.42, -0.58)	-4.68	2.91e-6	5.64e-5	Lower in brain
Ctla4	528.3 (39.7)	580.1 (85.2)	211.9 (48.0)	-0.14 (-0.45, 0.17)	-0.86	.390	.565	No evidence of differential expression
Itgae	32.0 (9.0)	70.1 (2.9)	306.1 (103.2)	-1.12 (-1.67, -0.58)	-4.07	4.64e-5	5.96e-4	Lower in brain
Cd69	877.5 (183.3)	433.9 (79.2)	1107.4 (130.4)	1.02 (0.71, 1.32)	6.47	9.70e-11	6.10e-9	Higher in brain
Cxcr6	732.0 (10.0)	800.2 (89.9)	381.4 (54.8)	-0.13 (-0.36, 0.10)	-1.11	.266	.442	No evidence of differential expression
Ccr5	2982.1 (146.9)	2029.0 (87.5)	641.9 (108.0)	0.56 (0.35, 0.76)	5.28	1.27e-7	3.72e-6	Higher in brain
Cxcr3	1426.1 (252.9)	1745.8 (285.7)	918.9 (41.7)	-0.29 (-0.55, -0.04)	-2.25	.025	.085	No evidence of differential expression
Tnf	293.8 (183.8)	107.6 (10.8)	91.0 (8.6)	1.45 (0.91, 1.99)	5.27	1.36e-7	3.94e-6	Higher in brain
Tnfsf10	1043.9 (194.0)	831.8 (65.6)	465.8 (31.4)	0.33 (0.11, 0.55)	2.97	.003	.018	Higher in brain
Tnfsf14	236.9 (48.1)	55.5 (7.6)	80.4 (10.7)	2.09 (1.74, 2.44)	11.66	2.10e-31	1.21e-28	Higher in brain
Lta	15.3 (8.3)	16.7 (5.2)	47.1 (12.5)	-0.15 (-0.95, 0.65)	-0.36	.716	.823	No evidence of differential expression
Ltb	627.4 (143.5)	932.7 (107.8)	1372.2 (80.0)	-0.57 (-0.82, -0.32)	-4.49	7.18e-6	1.21e-4	Lower in brain
Fasl[a]	781.2 (28.5)	301.7 (36.0)	83.7 (27.7)	1.37 (1.05, 1.70)	8.23	1.82e-16	3.07e-14	Higher in brain

Data are mean (SD) DESeq2 size factor-normalized counts. The primary unpaired negative binomial model included brain PbA (*n* = 3), spleen PbA (*n* = 5), and spleen mock (*n* = 5) samples; the reported contrast was brain PbA versus spleen PbA. Spleen mock samples contributed to model fitting and provide contextual expression values but were not included in the reported contrast. Positive log2 fold-change values indicate higher expression in brain-infiltrating cells. Confidence intervals are 95% Wald intervals. *P* values are from two-sided Wald tests; *q* values were adjusted using the Benjamini-Hochberg procedure across 20,077 tested genes. The primary analysis corresponds to Fig. 5D and excludes brain sample 825B. A sensitivity analysis including 825B (brain *n* = 4) preserved the direction of effect for all displayed genes and the enrichment of *Tnf* ($\log_2$ FC = 2.94, $q = 6.8 \times 10^{-6}$) and *Fasl* ($\log_2$ FC = 1.30, $q = 8.6 \times 10^{-10}$); full per-gene results are in Supplementary Data S1. Abbreviations: CI, confidence interval; FDR, false discovery rate; PbA, Plasmodium berghei ANKA; SD, standard deviation. a, Fasl was not displayed in Fig. 5D and is included here as an additional death ligand because it is discussed in the Results.

Table S4. Regulated cell death pathway enrichment ratios from subphenotype-stratified GWAS across eight MalariaGEN cohorts.

Cohort	Subtype	*n* cases	*n* controls	λ_GC pathway	λ_GC background	Enrichment ratio
Gambia	CM	848	2786	1.032	1.007	1.024
Gambia	SMA	504	2786	0.944	1.002	0.943
Gambia	OTHER	1309	2786	1.103	1.011	1.091
Kenya	CM	821	1738	1.355	1.021	1.327
Kenya	SMA	208	1738	1.005	1.016	0.989
Kenya	OTHER	700	1738	1.182	1.042	1.135
Malawi	CM	754	1498	0.970	0.998	0.971
Malawi	SMA	84	1498	1.042	1.015	1.027
Malawi	OTHER	622	1498	0.693	1.015	0.683
Ghana	CM	33	582	1.224	1.047	1.169
Ghana	SMA	46	582	1.146	1.056	1.086
Ghana	OTHER	338	592	1.072	1.022	1.049
Mali	CM	84	93	1.372	1.027	1.336
Mali	SMA	179	94	1.499	1.085	1.382
Mali	OTHER	118	92	1.128	1.050	1.074
Burkina Faso	CM	100	598	0.921	1.064	0.866
Burkina Faso	SMA	32	597	0.995	1.067	0.933
Burkina Faso	OTHER	655	604	1.015	1.056	0.961
Cameroon	CM	35	609	1.116	1.048	1.065
Cameroon	SMA	76	612	0.844	1.069	0.789
Cameroon	OTHER	573	617	1.134	1.023	1.108
Tanzania	CM	35	585	1.117	1.058	1.056
Tanzania	SMA	208	594	0.859	1.024	0.839
Tanzania	OTHER	213	592	0.969	1.020	0.949

Enrichment ratio = λ_GC(regulated cell death variants) / λ_GC(genome-wide background). Independent Firth logistic regression GWAS were performed for each subphenotype (CM, cerebral malaria; SMA, severe malarial anemia; OTHER, other severe malaria) versus population controls within each cohort. Data from MalariaGEN consortium *(48, 79)*.

Table S5. Reagents

Reagent type	Designation	Source	Catalog #	Clone	Dilution / Concentration	Application
Antibody	anti-CD3ε-APC	BioLegend	100312	145-2C11	1:100	Flow cytometry ($CD8^+$ T cell sorting)
Antibody	anti-CD8α-FITC	BioLegend	100706	53-6.7	1:100	Flow cytometry ($CD8^+$ T cell sorting)
Antibody	anti-CD44-PE-Cy7	BioLegend	103030	IM7	1:100	Flow cytometry ($CD8^+$ T cell sorting)
Antibody	TruStain FcX™ (anti-mouse CD16/32)	BioLegend	101320	93	1:100	Fc receptor blocking
Antibody	anti-mouse CD8α	BioXcell	BP0061	2.43	250 µg per mouse (i.p.)	In vivo $CD8^+$ T cell depletion
Antibody	Rat IgG2b isotype control (anti-KLH)	BioXcell	BE0090	LTF-2	250 µg per mouse (i.p.)	Isotype control (in vivo)
Antibody	anti-cleaved caspase 3 (cCASP3)	R&D Systems	MAB835	N/A	1:300	IHC (mouse brain); DAB
Antibody	anti-phospho-MLKL (pMLKL, S345)	Abcam	ab196436	N/A	1:2000	IHC (mouse brain); DAB
Antibody	rabbit anti-CD31	Novus Biologicals	NB100-2284	Polyclonal	0.05 µg mL^{-1}	IF (human tissue); Opal 690
Antibody	goat anti-fibrinogen	Invitrogen	PA1-26809	Polyclonal	4.5 µg mL^{-1}	IF (human tissue); Opal 570
Antibody	rabbit anti-phospho-RIPK1	Cell Signaling Technology	44590S	D8I3A	0.38 µg mL^{-1}	IF (human tissue); Opal 520
Antibody	ImmPRESS-HRP anti-rabbit IgG	Vector Laboratories	MP-7401	N/A	Per kit protocol	IF secondary (human tissue)
Antibody	ImmPRESS-HRP anti-goat IgG	Vector Laboratories	MP-7405	N/A	Per kit protocol	IF secondary (human tissue)
Nanobody	anti-GFP nanobody (GFP-Trap Magnetic Agarose)	ChromoTek	gtma-20	N/A	N/A	IP (TRAP-seq)
Blocking reagent	BLOXALL Endogenous Blocking Solution	Vector Laboratories	SP-6000-100	N/A	N/A	IF (human tissue)
Blocking reagent	FcR Blocking Reagent	Miltenyi Biotec	130-059-901	N/A	N/A	IF (human tissue)
Detection kit	Opal 7-Color IHC Kit	Akoya Biosciences	NEL811001KT	N/A	N/A	TSA detection (human IF)
Detection kit	Histofine Simple Stain MAX PO Universal immunoperoxidase polymer kit	Nichirei Biosciences	N/A	N/A	Per kit protocol	IHC detection; DAB
Counterstain	Hematoxylin	Ventana Roche	760-2021	N/A	4 min	IHC counterstaining
Counterstain	Bluing Reagent	Ventana Roche	760-2037	N/A	4 min	IHC bluing
Chemical	Durcupan ACM	Sigma-Aldrich	44610	N/A	Per protocol	TEM embedding
Chemical	Osmium tetroxide	Sigma-Aldrich	05500	N/A	0.5%	TEM fixation
Chemical	GNE684 (RIPK1 inhibitor)	Genentech	N/A (provided)	N/A	20 mg kg^{-1} (oral, b.i.d.)	In vivo RIPK1 inhibition
Chemical	Tamoxifen	Sigma-Aldrich	T5648	N/A	75 mg kg^{-1} (i.p.)	Cre recombination
Chemical	Cycloheximide	Sigma-Aldrich	C7698	N/A	100 µg mL^{-1}	Translation arrest (TRAP-seq)

Chemical	Puromycin dihydrochloride	Sigma-Aldrich	P8833	N/A	Per protocol	Endothelial cell selection
Chemical	Recombinant mouse IFN-γ	PeproTech	315-05	N/A	10 ng mL^{-1}	MHC-I induction (PMBECs)
Chemical	Albumin from chicken egg white (OVA)	Sigma-Aldrich	A5503	N/A	0.3 mg mL^{-1}	Antigen loading (in vitro)
Chemical	Recombinant mouse IL-2	PeproTech	212-12	N/A	50 U mL^{-1}	CTL culture medium
Chemical	Kaolin	Sigma-Aldrich	K7375	N/A	10 µg mL^{-1}	Coagulation activation
Lectin	Lycopersicon esculentum lectin-DyLight™ 649	Vector Laboratories	DL-1178	N/A	10 µg mL^{-1}	Vascular labeling (LSFM)
Reagent kit	Thrombin Calibrator	Thrombinoscope BV	TS20.00	N/A	N/A	CAT assay
Reagent kit	PPP-Reagent LOW (TF 1 pM + PL 4 µM)	Thrombinoscope BV	TS31.00	N/A	N/A	CAT assay
Reagent kit	MP-Reagent (PS:PC:PE 20:60:20)	Thrombinoscope BV	TS60.00	N/A	N/A	CAT assay
Reagent kit	FluCa-Kit (Z-Gly-Gly-Arg-AMC)	Thrombinoscope BV	TS50.00	N/A	N/A	CAT assay
Commercial kit	CellTiter-Glo® Luminescent Cell Viability Assay	Promega	G7570	N/A	N/A	Viability quantification
Commercial kit	RNeasy Micro Kit	Qiagen	74004	N/A	N/A	RNA purification (TRAP-seq)
Commercial kit	SMART-Seq v4 Ultra Low Input RNA Kit	Takara Bio	634888	N/A	N/A	cDNA library prep
Commercial kit	NEBNext Ultra II RNA Library Prep Kit	New England Biolabs	E7770	N/A	N/A	cDNA library prep

SUPPLEMENTARY AUXILIARY FILES

VIDEOS

Video S1. Axial SWI sequence demonstrating cerebral malaria microhemorrhages. Sequential axial slices from a representative patient showing multiple punctate hypointense lesions at the juxtacortical junction, scrolling from inferior to superior levels. This SWI sequence illustrates the characteristic distribution pattern of microbleeds quantified in **Fig. 1**.

Video S2. Three-dimensional LSFM visualization of hemorrhagic pathology in experimental cerebral malaria. Panoptic rendering of a cleared whole brain from a PbA-infected mouse at day six post-infection. Brain vasculature is labeled with tomato lectin (white/red), revealing extensive hemorrhagic lesions throughout the brain. The 360-degree rotation demonstrates the spatial distribution of hemorrhages, with prominent involvement of the olfactory bulb and posterior brain regions. This LSFM scan represents the raw data used for the automated hemorrhage quantification presented in **Fig. 2**.

Video S3. High-magnification LSFM visualization of perivascular hemorrhagic patterns. Zoomed three-dimensional rendering of hemorrhagic foci in the olfactory bulb of a PbA-infected mouse. The video shows characteristic ring hemorrhages with radial extravasation extending from disrupted microvessels into the parenchyma. Individual erythrocytes can be seen dispersing through compromised vessel walls, forming distinct perivascular hemorrhagic zones. These patterns correspond to the perivascular microbleeds (cluster 0) and confluent bleeding zones (cluster 3) identified in **Fig. 2**.

Video S4. Three-dimensional visualization of hemorrhage segmentation results. Three-dimensional rendering of the neural-network-based hemorrhage segmentation pipeline applied to LSFM data from a PbA-infected mouse. Color-coded hemorrhagic regions represent the four clusters identified by *k*-means classification: perivascular microbleeds (cluster 0, cyan), diffuse hemorrhagic zones (cluster 1, gray), punctate hemorrhages (cluster 2, pink), and confluent bleeding (cluster 3, orange). A 360-degree rotation of the spatial distribution and volumetric quantification of the segmented hemorrhages shown in **Fig. 2H** is presented.

DATA

Data S1. Differential-expression statistics and normalized counts underlying Fig. 5D, including the sensitivity analysis with brain sample 825B. Gene-level differential-expression results and DESeq2 size factor-normalized counts for the $CD8^+$ T-cell RNA-sequencing analysis. The reported comparison is brain-infiltrating versus splenic $CD8^+$ T cells from *Plasmodium berghei* ANKA (PbA)-infected mice. The primary analysis excludes brain sample 825B; the sensitivity analysis includes this sample. Mock-infected spleen samples contributed to model fitting but were not part of the reported contrast. Positive log2 fold-change values indicate higher expression in brain-infiltrating cells. *P* values derive from two-sided Wald tests; adjusted *P* values were calculated using the Benjamini–Hochberg procedure. Normalized counts represent sequencing counts adjusted by DESeq2 size factors, rather than absolute transcript abundance. Log2 fold changes and P values are dimensionless. Abbreviations: PbA, *Plasmodium berghei* ANKA; FC, fold change; FDR, false discovery rate.
